# Link clustering explains non-central and contextually essential genes in protein interaction networks

**DOI:** 10.1038/s41598-019-48273-3

**Published:** 2019-08-12

**Authors:** Inhae Kim, Heetak Lee, Kwanghwan Lee, Seong Kyu Han, Donghyo Kim, Sanguk Kim

**Affiliations:** 1 Department of Life Sciences, Pohang University of Science and Technology, Pohang, 37673 Korea; 2 School of Interdisciplinary Bioscience and Bioengineering, Pohang University of Science and Technology, Pohang, 37673 Korea

**Keywords:** Robustness, Network topology

## Abstract

Recent studies have shown that many essential genes (EGs) change their essentiality across various contexts. Finding contextual EGs in pathogenic conditions may facilitate the identification of therapeutic targets. We propose link clustering as an indicator of contextual EGs that are non-central in protein-protein interaction (PPI) networks. In various human and yeast PPI networks, we found that 29–47% of EGs were better characterized by link clustering than by centrality. Importantly, non-central EGs were prone to change their essentiality across different human cell lines and between species. Compared with central EGs and non-EGs, non-central EGs had intermediate levels of expression and evolutionary conservation. In addition, non-central EGs exhibited a significant impact on communities at lower hierarchical levels, suggesting that link clustering is associated with contextual essentiality, as it depicts locally important nodes in network structures.

## Introduction

A gene is essential for cell viability when its loss-of-function is lethal to the cell. Gene essentiality is contextual, as a gene can change its essentiality across different species, growth media, and chemical treatments^1–6^. There is growing evidence that some essential genes (EGs) are more contextual than others. For instance, some yeast EGs were more evolvable (i.e., able to become non-essential) than others under laboratory conditions^7^. In addition, genome-scale loss-of-function screening across cancer cell lines revealed that a subset of genes were less often essential than others^8–10^. The identification of such contextual EGs holds great potential for the development of therapies that selectively suppress pathogenic cells by targeting genes that are essential in the pathogenic cells but not in healthy cells^11^.

**Figure 1:**
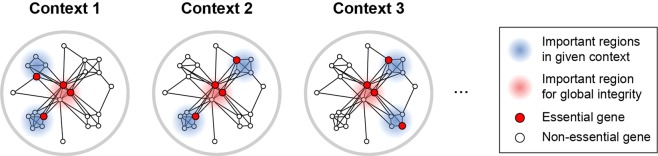
Hypothesis about the relationship between network structure and contextual essentiality. Non-central EGs might be essential in certain contexts, whereas central EGs would be essential for their role in global integration regardless of context.

Several reports have shown that invariable EGs are likely to be central nodes in protein-protein interaction (PPI) networks, whereas contextual EGs are less central. For instance, human genes that were broadly essential across cell lines showed greater degree than other genes in PPI networks^10,12^. In addition, yeast genes that remained essential during laboratory evolution showed greater degree than those that became non-essential^7^. An intuitive explanation for those observations is that central EGs are indispensable for their implication on global network integrity, which is imperative regardless of varying contexts that alter the pertinence of local regions (Fig. 1, red area). By contrast, non-central EGs can be indispensable in one context but dispensable in another context (Fig. 1, blue area). One implication of that hypothesis is that the characterization of non-central EGs would lead to the identification of contextual EGs.

Network clustering is a property that can characterize non-central EGs distinctly from central EGs. Previous studies showed that EGs tend to be directly connected to one another^13,14^ or enriched within the same functional modules and protein complexes^15–20^, thus making up essential modules. It has not been tested whether such clustered EGs are contextual. In addition, the question of whether a clustering measure could separate non-central EGs from central ones also needs to be explored. The nodes that make up essential modules tend to have greater degree than the nodes in other modules^20^. Furthermore, essential modules themselves have greater numbers of connections than non-essential modules in module-level networks^21^. A few studies have looked specifically at EGs with high link clustering^22,23^, but they tested the distinction between non-central EGs and central EGs only weakly by comparing 100 genes with highly ranked topology measures.

We hypothesize that highly clustered links, rather than merely a clustered network structure, contribute to gene essentiality, because such links represent functional dependency between nodes. It was previously proposed that links with stronger functional dependency have a greater impact on network robustness than links with weaker functional dependency, as the failure of one node with strong functional dependency will likely result in the failure of the whole neighborhood^24,25^. At the molecular level, obligate interactions among proteins are one example of functional dependency: a protein is unstable on its own, so it has to be bound to its partner to sustain its stability^26^. It has been shown that various link clustering measures can estimate functional dependency between nodes^27–29^.

We aimed to characterize the relationship between contextual EGs and non-central EGs. We systematically compared various clustering measures for their ability to characterize non-central EGs and investigated their association with contextual EGs. We found that link clustering is an accurate indicator of node essentiality independent of centrality, enabling us to correctly classify a substantial number of non-central EGs. EGs with clustered links were likely to change their essentiality across human cell lines and between species and, furthermore, showed levels gene expression and evolutionary conservation that were between those of central EGs and non-EGs. Moreover, the non-central EGs had profound impacts on communities at low-level hierarchy, supporting our hypothesis that network clustering is relevant to contextual essentiality because it characterizes locally pertinent nodes in the network.

## Results

### Centrality and link clustering characterize distinct facets of gene essentiality

Our goal was to find a clustering measure that is capable of characterizing EGs that are distinct from central EGs, which we classify as non-central EGs (see Fig. S1 for a flowchart). We investigated a node clustering measure (node clustering coefficient, *C*) and three link clustering measures (the product of two end nodes’ *C* values, *CXC*; the link clustering coefficient, *LCC*; and the edge clustering coefficient, *ECC*). Because gene essentiality is a property of nodes rather than of links, for each node we aggregated the link clustering measures with the node’s neighbors in PPIs by taking the average (*μCXC*, *μLCC*, and *μECC*) and the sum (*ΣCXC*, *ΣLCC*, and *ΣECC*). We compared those clustering measures to four centrality measures (degree, *DC*; betweenness, *BC*; closeness, *CC*; and eigenvector, *EC*) in eight different PPI networks in yeast and human. The details of the topology measures and PPI networks are further described in the *Supplementary Information* (*SI*).

**Figure 2:**
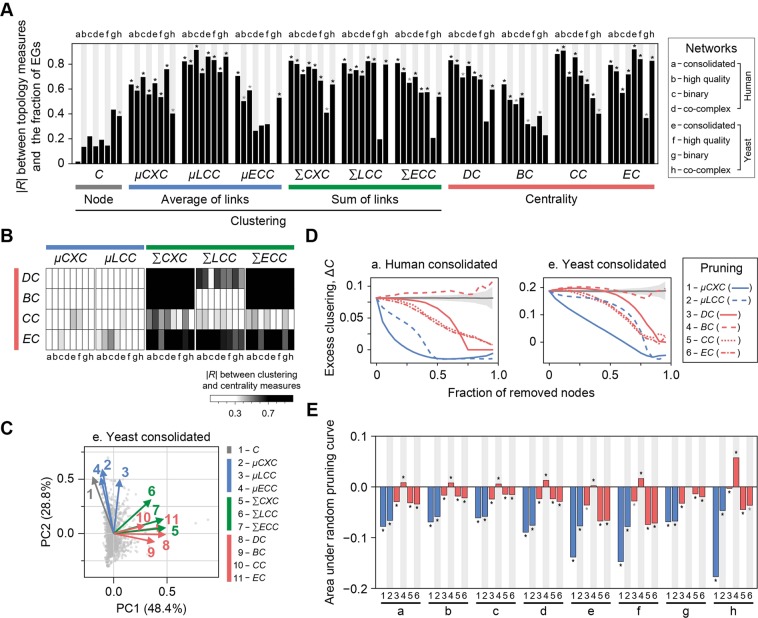
Relationships between topology measures and gene essentiality in PPI networks. (**A**) The absolute value of the Pearson correlation coefficient (|*R*|) between the fraction of EGs and topology measures. Proteins were sorted by a given topology measure and divided into bins each containing 2% of the population. (**B**) |*R*| between centrality measures and clustering measures. (**C**) Principle component (PC) analysis of topology measures on EGs in the yeast consolidated network. The variance explained by each component is given in parentheses. (**D**) Pruning analysis. The change in excess clustering (Δ*C*) was monitored while proteins were progressively removed in decreasing order of a given topology measure, or randomly (gray line; area, 3σ). Δ*C* is the difference between the observed *C* and the mean *C* of randomized networks. (**E**) Summary of pruning analyses in different networks. The decrease of Δ*C* was quantified by the area under the random pruning curve.

We found that several of the link clustering measures are as capable of characterizing gene essentiality as the centrality measures are (Fig. 2A). For each topology measure, we divided proteins into rank-ordered bins and calculated Pearson’s correlation coefficient (*R*) between the given measure and the fraction of EGs (*f*_E_). Five link clustering measures, including *μCXC*, *μLCC*, *ΣCXC*, *ΣLCC*, and *ΣECC*, showed significant correlations with *f*_E_ in most of the PPI networks, whereas *C* and *μECC* often failed to exhibit significant correlations (Fig. 2A; gray *P < 0.05; black *P < 0.001). In addition, we confirmed that the centrality measures (*DC*, *BC*, *CC*, and *EC*) also exhibited significant correlations with *f*_E_ in various PPI networks, which is consistent with many previous reports.

The average link clustering measures (*μCXC* and *μLCC*) were more distinct from the centrality measures than the sum measures (*ΣCXC*, *ΣLCC* and *ΣECC*), although they were all correlated with *f*_E_. We found that *μCXC* and *μLCC* mostly exhibited no correlation with the centrality measures (Fig. 2B; blue versus red), whereas *ΣCXC*, *ΣLCC*, and *ΣECC* were often strongly correlated with the centrality measures (Fig. 2B; green versus red). To explore the relationship among topology measures more comprehensively, we conducted a principal component analysis (PCA) of EGs. In the yeast consolidated network, the average link clustering measures (Fig. 2C; 2–4, blue arrows) were roughly orthogonal to the centrality measures (Fig. 2C; 8–11, red arrows), whereas the sum link clustering measures (Fig. 2C; 5–7, green arrows) were prominently oriented in a similar direction with the centrality measures. We observed similar results in other PPI networks (Fig. S2). Therefore, the sum measures were not suitable for our goal to find non-central EGs, because they seemed to depict central EGs. All of the correlations between topology measures and gene essentiality, and their statistical significance, are shown in Table S1.

Because *µCXC* and *µLCC* were correlated with *f*_E_ (Fig. 2A) and not with the centrality measures (Fig. 2B,C), we examined them further for distinction from the centrality measures. We conducted a pruning analysis in which we removed nodes in decreasing order of a given topology measure and monitored the resultant change in excess clustering (Δ*C*), the difference between the observed *C* and the average *C* of random networks subjected to degree sequence-preserved randomization. The pruning analysis provided a comparison of topology measures in terms of their implication on network clustering.

We found that *µCXC* had greater implication on network clustering than *µLCC* and, more importantly, was more distinct from the centrality measures than *µLCC*. In the human consolidated network, for instance, the pruning curve of *µCXC* exhibited a slightly faster decrease of Δ*C* than that of *µLCC* (Fig. 2D, left; line 1 versus line 2), indicating that *µCXC* had a somewhat stronger impact on network clustering. In that case, both link clustering measures were distinguishable from the centrality measures, as the centrality measures showed much slower decreases of ∆*C* (Fig. 2D, left; lines 3–6). By contrast, in the yeast consolidated network, the pruning curve of *µLCC* (Fig. 2D, right; line 2) overlapped those of two centrality measures, *CC* and *EC* (Fig. 2D, right; lines 5 and 6, respectively), indicating that those three topology measures had similar impacts on network clustering. We quantified the decrease of ∆*C* by measuring the area over the curve of each parameter and under that of random pruning (Fig. 2D, gray line), in which proteins were removed in a random order. We observed that *µCXC* was distinct from the centrality measures in all the PPI networks, whereas *µLCC* was similar to the centrality measures in the yeast consolidated, high-quality, and co-complex networks (Fig. 2E; see Fig. S3 for all the pruning curves).

Since *µCXC* was the most distinct from the centrality measures and capable of characterizing gene essentiality, we used it to classify non-central EGs. For the sake of simplicity, we refer to *µCXC* as *w* throughout the rest of the manuscript, as it represents the link weights. We selected *DC* as a counterpart to classify central EGs and refer to it as *k*.

### Link clustering characterizes a distinct subset of non-central EGs

Given that gene essentiality can be characterized by two uncorrelated properties, *k* and *w*, we expect EGs to fall into two distinct subsets: those better characterized by *k* (*k*-dependent) and those better characterized by *w* (*w*-dependent). Using logistic regression, we calculated the probabilities of being essential based on *k*, *P*_E_(*k*), and based on *w*, *P*_E_(*w*). We then classified EGs as *k*-dependent if *P*_E_(*k*) > *P*_E_(*w*) or as *w*-dependent if *P*_E_(*k*) < *P*_E_(*w*) (Fig. S4). Considering the cases where EGs are explained by neither *k* nor *w*, we discarded EGs under cutoffs k_c_ and w_c_, which maximized Matthew’s correlation coefficient (MCC) by regarding only genes with *k* ≥ k_c_ or *w* ≥ w_c_ as predicted EGs (Fig. S5).

**Figure 3:**
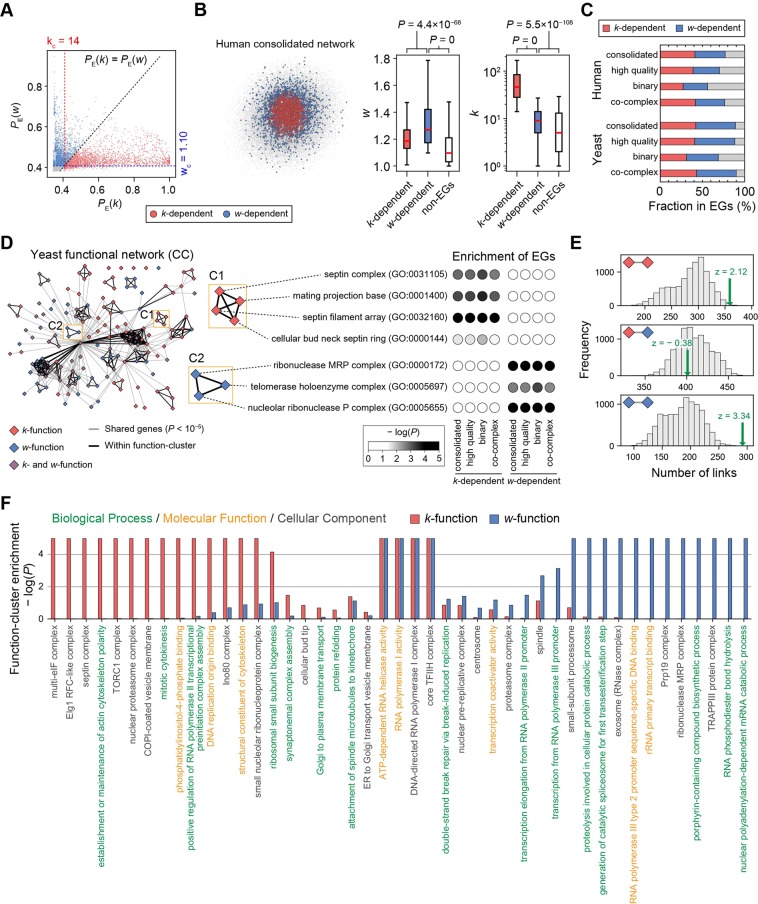
Classification of *k*-dependent and *w*-dependent EGs and their functional differences. (**A**) Classification of *k*-dependent and *w*-dependent EGs based on the probability of being essential (*P*_E_) inferred from logistic regression with *k* and *w*. Cutoffs (k_c_ and w_c_) were determined to maximize MCC for each topology measure, and proteins under the cutoffs remained unclassified. (**B**) (left) *k*-dependent and *w*-dependent EGs in the network. (right) Topological differences among classified EGs and non-EGs. (**C**) Fraction of *k*-dependent and *w*-dependent EGs in different PPI networks. (**D**) The functional network of yeast “cellular component” (CC) terms connected by shared genes. Terms were identified as *k*-functions or *w*-functions when they were enriched with *k*-dependent or *w*-dependent EGs, respectively, in three or four PPI networks. Function-clusters were determined by the MCODE algorithm. (**E**) Number of links between *k*-functions and *w*-functions in the functional network of yeast CC terms; (upper) between two *k*-functions; (middle) between a *k*-function and a *w*-function; (lower) between two *w*-functions. Green arrows indicate the observed number in the real network, and gray bars show the number distribution in 10,000 random sets with shuffled *k*-functions and *w*-functions. (**F**) Representative GO terms of function-clusters from yeast functional networks and the bias of clusters toward *k*-functions and *w*-functions. *P*-values < 10^−5^ were set to 10^−5^.

We found that a sizable number of EGs were *w*-dependent. In the human consolidated network, 36.0% of EGs were *w*-dependent (*n* = 2,186; Fig. 3A, left; blue circles), which is comparable to the proportion of *k*-dependent EGs (40.9%, *n* = 2,483; Fig. 3A, left; red circles). Those two subsets of EGs were very distinctive in the network structure (Fig. 3B, left). As expected, the *w*-dependent EGs showed greater *w* than the *k*-dependent EGs (Fig. 3B, right; *P* = 4.4 × 10^−68^, Mann-Whitney U [MWU] test) and the non-EGs (*P* = 0), and they had intermediate *k* compared with the *k*-dependent EGs (*P* = 0) and the non-EGs (*P* = 5.5 × 10^−108^). In the eight different PPI networks, 29–47% of EGs were *w*-dependent (Fig. 3C). All of the *k*-dependent and *w*-dependent EGs in different PPI networks are shown in Tables S3, S4 for yeast and human, respectively.

We next examined whether *k*-dependent and *w*-dependent EGs are distinct with respect to not only network structure but also biological function. We defined *k*-functions and *w*-functions as gene ontology (GO) terms enriched with *k*-dependent and *w*-dependent EGs, respectively, in three or four PPI networks. Because different GO terms could be similar to each other, we constructed functional networks of GO terms connected by shared genes and investigated function-clusters in which GO terms were densely connected (see the *Methods*).

We found that *k*-dependent and *w*-dependent EGs were associated with distinct biological functions. In the yeast functional network composed of Cellular Components (CC) terms, many function-clusters were biased toward either *k*-functions or *w*-functions (Fig. 3D; see Fig. S6 for all the functional networks). For instance, one function-cluster was composed of four similar *k*-functions (“septin complex”, “mating projection base”, “septin filament array”, and “cellular bud neck septin ring”) that were enriched with *k*-dependent EGs from all four yeast PPI networks (Fig. 3D; box C1). Another cluster possessed three related *w*-functions (“ribonuclease MRP complex”, “telomerase holoenzyme complex”, and “nucleolar ribonuclease P complex”) that were enriched with *w*-dependent EGs from all four yeast PPI networks (box C2). In addition, links in the functional network were observed more frequently between pairs of *k*-functions (Fig. 3E, upper panel; *n* = 359, *z* = 2.12) and between pairs of *w*-functions (Fig. 3E, lower panel; *n* = 292, *z* = 3.34) compared with those observed in 10,000 random sets with shuffled *k*-function and *w*-function tags. By contrast, links between *k*-functions and *w*-functions were observed at a frequency similar to the random expectation (Fig. 3E, middle panel; *n* = 401, *z* = −0.38). That result was robust over all functional networks composed of three GO categories in yeast and human (Fig. S7).

We made a comprehensive summary of the biological functions associated with *k*-dependent and *w*-dependent EGs (Fig. 3F for yeast; Fig. S8 for human). We selected a GO term with the median size, as determined by the number of genes assigned to the term, as a representative for each function-cluster. Among 45 function-clusters in the yeast functional networks, only four (“ATP-dependent RNA helicase activity”, “RNA polymerase I activity”, “DNA-directed RNA polymerase II, core complex”, and “core TFIIH complex”) were biased toward both *k*-functions and *w*-functions (−log[*P*] ≥ 2, hypergeometric test). By contrast, 15 and 14 function-clusters were biased toward either *k*-functions or *w*-functions, respectively. Function-clusters biased toward *k*-functions often represented cytokinesis (e.g., “septin complex”, “establishment or maintenance of actin cytoskeleton polarity”, and “mitotic cytokinesis”), whereas those biased toward *w*-functions corresponded to RNA degradation (e.g., “exosome [RNase complex]”, “ribonuclease MRP complex”, and “nuclear polyadenylation-dependent mRNA catabolic process”). Taken together, those results demonstrate that link clustering characterizes a unique subset of EGs with distinct biological functions. All *k*-functions and *w*-functions and their clusters in yeast and human are shown in Tables S5 and S6, respectively.

### *w*-dependent EGs are more contextual than *k*-dependent EGs

There is growing evidence that gene essentiality is often contextual, meaning that a gene may change its essentiality across cell lines and species. Given that central genes tend to be evolutionarily conserved and expressed broadly across cell lines, we expect that *w*-dependent EGs might be prone to change their essentiality, as they are less central than *k*-dependent EGs.

**Figure 4:**
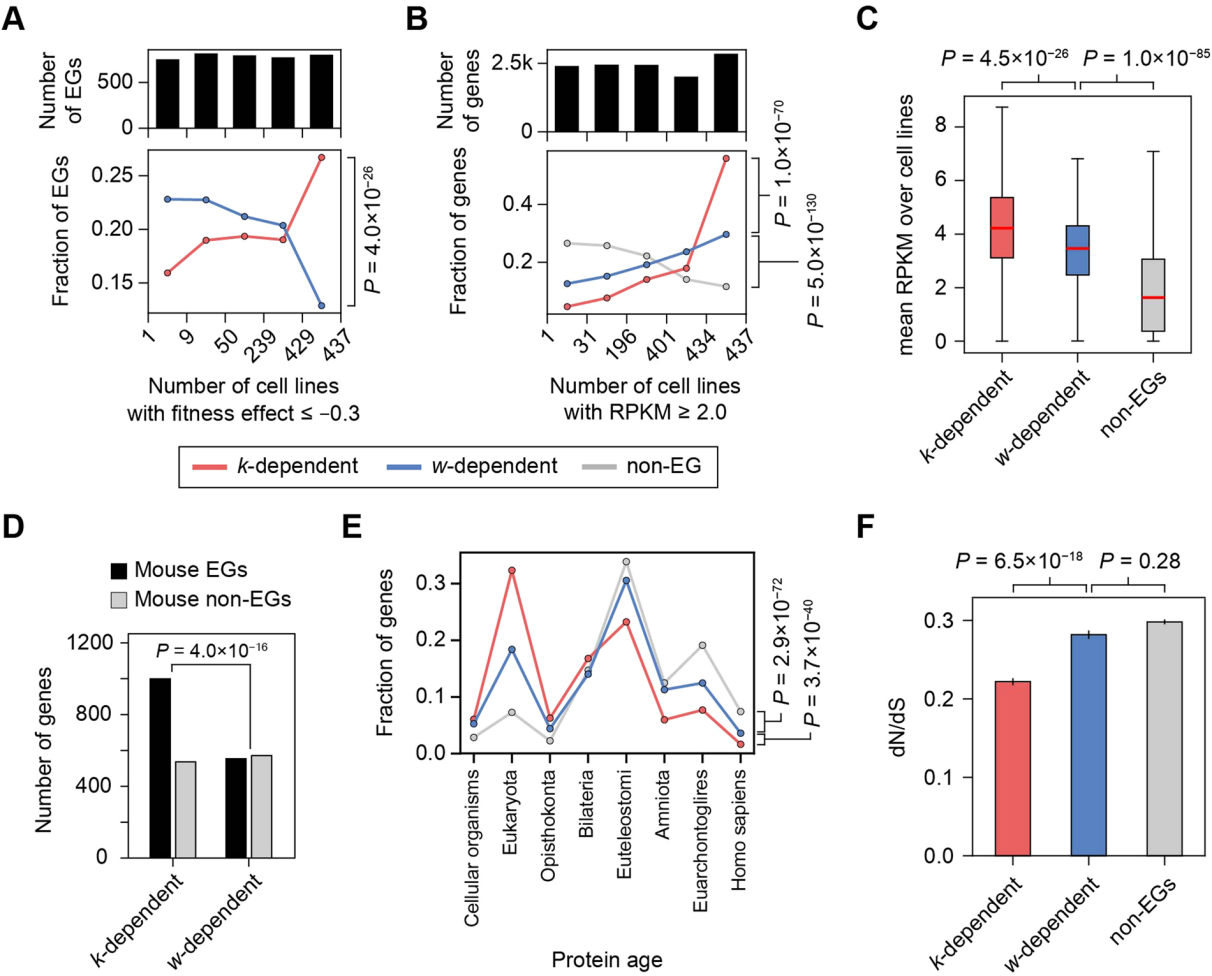
Contextual essentiality of human *k*-dependent and *w*-dependent EGs. (**A**) Contextual gene essentiality across human cell lines for *k*-dependent and *w*-dependent EGs. Bins were divided to have similar populations of EGs. (**B**) Contextual gene expression across human cell lines. Bins were divided to have similar populations of all genes. (**C**) Expression levels of genes across human cell lines. (**D**) Essentiality change of *k*-dependent and *w*-dependent EGs between mouse and human. (**E**) Phyletic age and (**F**) evolutionary rate (dN/dS) of *k*-dependent and *w*-dependent EGs and non-EGs.

As expected, we found that the essentiality of *w*-dependent EGs was more cell-line-specific than that of *k*-dependent EGs (Fig. 4A). Using a publicly available dataset of genetic vulnerability screens in 436 cancer cell lines, we measured the broadness of essentiality for each gene as the number of cell lines in which the given gene exhibited a fitness-effect ≤ −0.3. We then divided the EGs into five mostly even bins and observed the distributions of *k*-dependent and *w*-dependent EGs. In the human consolidated network, the *w*-dependent EGs tended to be essential in a relatively small number of cell lines, whereas the *k*-dependent EGs were essential in a greater number of cell lines (Fig. 4A; *P* = 4.0 × 10^−26^, χ^2^ test). Supporting that observation, we also found that *w*-dependent EGs exhibited an intermediate level of expression between those of *k*-dependent EGs and non-EGs (Fig. 4B,C). With the expression dataset matched to the genetic vulnerability screens, *w*-dependent EGs tended to be expressed less broadly among the cell lines than *k*-dependent EGs (Fig. 4B, *P* = 1.0 × 10^−70^) and more broadly than non-EGs (*P* = 5.0 × 10^−130^). In addition, the average expression level of *w*-dependent EGs was lower than that of *k*-dependent EGs (Fig. 4C, *P* = 4.5 × 10^−26^) and higher than that of non-EGs (*P* = 1.0 × 10^−85^). We observed similar results in other PPI networks with different cutoffs (Figs S9–11), except in the binary network.

We also found that the essentiality of *w*-dependent EGs was more frequently changed between human and mouse than that of *k*-dependent EGs. In the human consolidated network, mouse orthologs of *w*-dependent EGs were more frequently identified as non-essential (Fig. 4D; fraction = 50.8%) than those of *k*-dependent EGs (35.0%, *P* = 4.0 × 10^−16^, Fisher’s exact test), indicating that the essentiality of *w*-dependent genes was less conserved than that of *k*-dependent EGs. We also found that *w*-dependent EGs exhibited an intermediate level of molecular conservation compared with *k*-dependent EGs and non-EGs (Fig. 4E,F). We estimated protein ages on the basis of a reconstructed history of protein families and found that *w*-dependent EGs were younger than *k*-dependent EGs (Fig. 4E; *P* = 3.7 × 10^−40^, χ^2^ test) and older than non-EGs (*P* = 2.9 × 10^−72^). In addition, we observed that the evolutionary rate (dN/dS, ratio of synonymous to non-synonymous nucleotide substitutions) of *w*-dependent EGs was greater than that of *k*-dependent EGs (Fig. 4F; *P* = 6.5 × 10^−18^, MWU test) and less than that of non-EGs, although the latter difference was not statistically significant. Similar results were observed in other PPI networks with different cutoffs (Figs S12, S13), except in the binary network.

Taken together, the results strongly suggest that *w*-dependent EGs are more contextual than *k*-dependent EGs. The investigated molecular properties, such as gene expression and evolutionary conservation, only characterized *w*-dependent EGs as analogous to non-EGs (Fig. 4B,C,E,F), whereas the link clustering showed *w*-dependent EGs as being further apart from non-EGs than from *k*-dependent EGs (Fig. 3B).

### *w*-dependent EGs significantly impact communities at low levels of hierarchy

Because many previous studies have already suggested network clustering as a property pertinent to gene essentiality, we examined *k*-dependent and *w*-dependent EGs more precisely regarding the clustered network structure around them. Specifically, we found that *k*-dependent EGs are well-clustered in a generic sense, whereas *w*-dependent EGs are specifically relevant to network communities at low levels of hierarchy.

**Figure 5:**
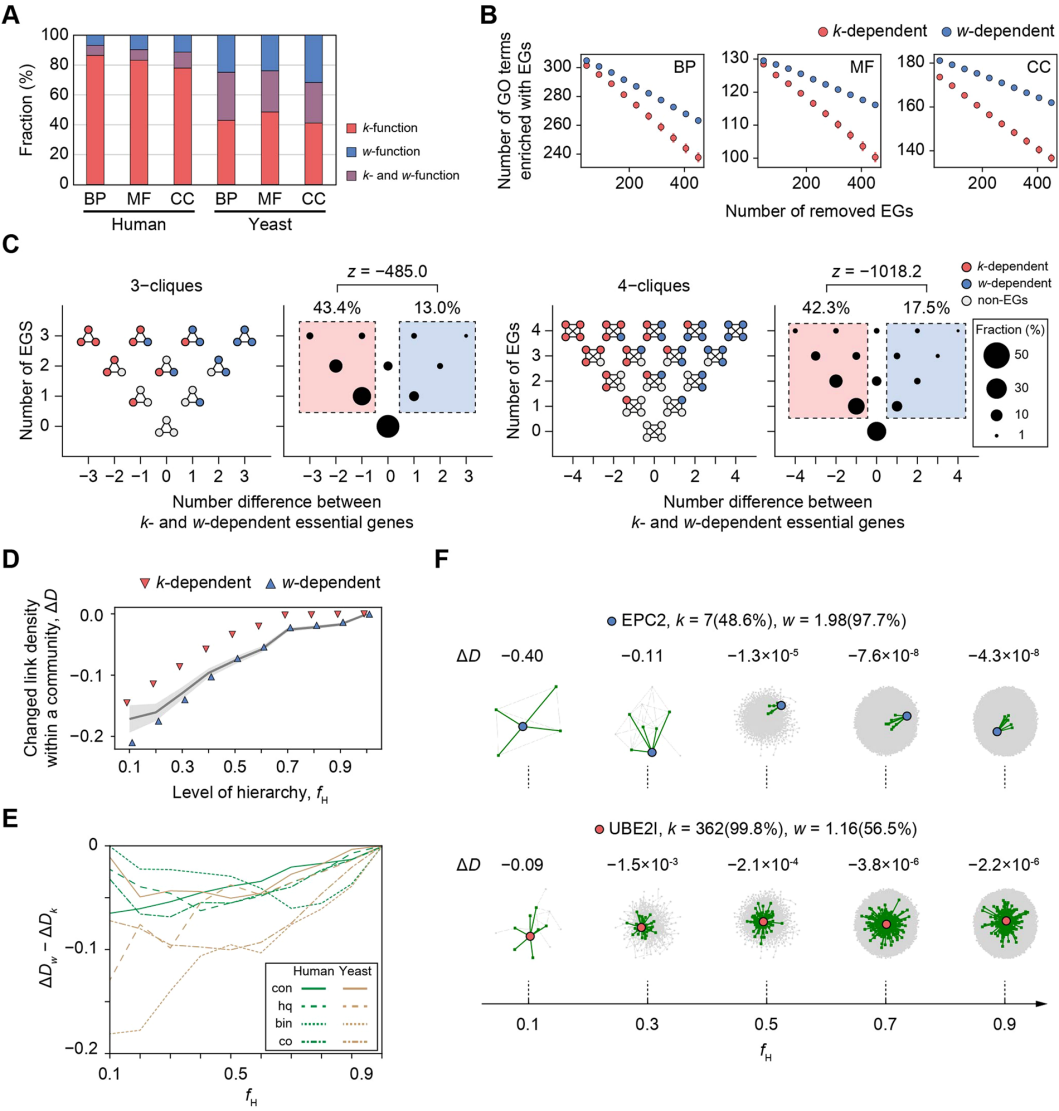
Clustering of *k*-dependent and *w*-dependent EGs. (**A**) Relative frequency of GO terms enriched with *k*-dependent and *w*-dependent EGs. (**B**) Decrease of enriched GO terms due to removal of the same number of *k*-dependent or *w*-dependent EGs. (**C**) *n*-cliques and their biases toward *k*-dependent and *w*-dependent EGs. The normal distribution of *z* values was approximated by binomial trials with success probability given by the fraction of cliques biased toward *k*-dependent EGs. (**D**) Change in the link density of a community upon removal of a single node (Δ*D*) at different hierarchical levels (*f*_H_). Hierarchical community structure was searched using the *Walktrap* algorithm. The gray line indicates the average Δ*D* for non-EGs (area, standard error). (**E**) Difference in Δ*D* between *k*-dependent and *w*-dependent EGs in different PPI networks. (**F**) Examples illustrating *EPC2*, a *w*-dependent EG, and *UBE2I*, a *k*-dependent EG, for their impact on communities. The protein of interest (blue circles, *EPC2*; red circles, *UBE2I*) and its interactions (green lines) with its first neighbors (green circles) are shown in the community. Numbers in parentheses indicate the rank percentile for *k* and *w*.

We found that *k*-dependent EGs were more clustered than *w*-dependent EGs (Fig. 5A–C). There were more *k*-functions than *w*-functions (Fig. 5A), indicating that *k*-dependent EGs are more likely to be clustered into the same functions than *w*-dependent EGs. Because the difference in the numbers of *k*-dependent and *w*-dependent EGs might affect the observed enrichment, we randomly removed the same number of EGs from each category and monitored the decrease of enriched GO terms. In the yeast consolidated network, for instance, the removal of *k*-dependent EGs lead to a greater decrease in the number of enriched GO terms than the removal of *w*-dependent EGs in all three GO categories (Fig. 5B). To further examine the clustered network structure around EGs, we searched *n*-cliques, which are fully connected subgraphs with *n* nodes, and investigated their bias toward *k*-dependent or *w*-dependent EGs. We found that cliques frequently included more *k*-dependent EGs than *w*-dependent EGs (Fig. 5C; 3-cliques, fraction = 43.4% versus 13.0%, *z* = −485.0; 4-cliques, fraction = 42.3% versus 17.5%, *z* = −1018.2). Similar results were observed in other PPI networks, except in the binary networks (Figs S14–S15). Those results indicate that *k*-dependent EGs are densely clustered into the same biological functions, possibly because of their greater number of links.

The observed clustering of *k*-dependent EGs raises the question of how the link clustering measure, *w*, separates a subset of non-central EGs. Given their contextual essentiality, we hypothesized that *w*-dependent EGs would significantly impact small communities at low levels of hierarchy, because a system’s dependency on a small and local community would be more context-specific than that on a large and global community. To test that hypothesis, we investigated the impact of the removal of a node by monitoring changes of link density, Δ*D*, in communities at various hierarchical levels, *f*_H_, defined by the fraction of prior merges in the agglomerative clustering (see the *Methods*).

We found that the impact on link density within a community was greater for the removal of *w*-dependent EGs than for that of *k*-dependent EGs, and the difference was significant at lower hierarchical levels. In the human consolidated network with *f*_H_ = 0.1, we observed a greater decrease in link density for *w*-dependent EGs (Fig. 5D, Δ*D*_*w*_ = −0.210) than for *k*-dependent EGs (Δ*D*_*k*_ = −0.145) upon removal of a single node, suggesting that *w*-dependent EGs have a greater impact on community structure. By contrast, the difference in Δ*D* between *k*-dependent and *w*-dependent EGs became extremely small at the highest hierarchical level (Δ*D*_*w*_ − Δ*D*_*k*_ = −0.00052, *f*_H_ = 1.0), suggesting that the effect of a single node removal is unlikely to be distinguishable at the level of the global network. Additionally, to confirm that Δ*D* is relevant to gene essentiality, we looked at changes in link density upon the removal of a single node for non-EGs (Δ*D*_*n*_). At lower hierarchical levels (*f*_H_ ≤ 0.4), Δ*D*_*w*_ − Δ*D*_*n*_ < 0, indicating that *w*-dependent EGs had a greater impact on local community structure than non-EGs. Similar results were observed in other PPI networks; Δ*D*_*w*_ − Δ*D*_*k*_ < 0 (Fig. 5E) and Δ*D*_*w*_ − Δ*D*_*n*_ < 0 (Fig. S17) at lower hierarchical levels with low *f*_H_ values.

An example of the impact of single node deletions on community structure with varying hierarchy is shown in Fig. 5F. At *f*_H_ = 0.1, the deletion of *EPC2*, a *w*-dependent EG with few and clustered links (*k* = 7 [rank percentile = 48.6%], *w* = 1.98 [rank percentile = 97.7%]) had a large impact on the community structure (Δ*D* = −0.40), causing the removal of four of nine total links. By contrast, the deletion of *UBE2I*, a *k*-dependent EG with many unclustered links (*k* = 362 [rank percentile = 99.8%], *w* = 1.16 [rank percentile = 56.5%]) had a smaller impact on the community structure (Δ*D* = −0.09), although more links (*n* = 7) were removed. At higher hierarchical levels (*f*_H_ ≥ 0.5), both *EPC2* and *UBE2I* became members of the same large communities, so the impact of their deletion was much smaller (Δ*D*_EPC2_ = −1.3 × 10^−5^ and Δ*D*_UBE2I_ = −2.1 × 10^−4^, at *f*_H_ = 0.5) than at lower levels of hierarchy. Taken together, those results indicate that both *k*-dependent and *w*-dependent EGs could be considered “clustered” in some sense, whereas the link clustering discretely characterizes EGs that are crucial for communities at low levels of hierarchy.

## Discussion

We demonstrated that a link clustering measure, *w*, is capable of characterizing non-central and contextual EGs. For the understanding of contextual gene essentiality, the biological significance of link clustering measures remains a matter of scientific exploration. Our results strongly suggest that functional dependency between nodes, rather than network clustering per se, is crucial for depicting contextual EGs. We observed that *w*-dependent EGs have distinct implications on communities at low levels of hierarchy (Fig. 5D–F), in which strong functional relevance among member nodes is expected. Recent reports showed that a gene’s essentiality across varying contexts is largely dependent on its neighbors with strong functional relevance^30–32^. Moreover, links conveying strong functional dependency may have a significant impact on network robustness, as the failure of one node will likely cascade over them^24,25^. Taken together, our results suggest that the link clustering measure *w* estimates functional dependency between two nodes and portrays genes that are functionally pivotal to their neighbors in non-central regions of cellular systems.

Many previous studies suggested relevance between gene essentiality and network clustering, so one might reasonably ask whether non-central EGs were characterized in those studies^13–20^. It is worth noting that “clustering” is a general concept of network structure, and a clustering measure may or may not distinguish non-central nodes from central ones. In our dataset, we observed that some clustering measures, other than *w*, were correlated with centrality measures (Fig. 2A–C), and that central EGs were in some sense “clustered” with respect to functional modules and network cliques (Fig. 5A–D). Therefore, in working toward the goal to separate non-central EGs from central EGs, one needs to carefully assess different topology measures, as each measure characterizes a distinct facet of the network structure.

The limitation of our work is that the link clustering measure *w* is incapable of estimating a gene’s essentiality for a given context, despite its ability to characterize the tendency for a gene to be contextual across contexts. Precise estimation of gene essentiality for a given cell line has potential for the development of therapeutic targets that specifically eliminate pathogenic cells without causing excessive damage to normal cells^9,10^. In addition, recent genome-scale fitness screens enabled the identification of molecular biomarkers for contextual essentiality, providing insights into the molecular mechanisms underlying the vulnerability of pathogenic cells^33,34^. We anticipate that the further classification of EGs will provide a useful indication of varying gene essentiality in different contexts.

It has been argued that disease genes are devoid of essentiality and network centrality, because the impairment of an EG would likely cause the death of the organism rather than manifest disease phenotypes^35^. Associations between EGs and diseases might be more prevalent than expected^36,37^, however, because many genetic perturbations that occur naturally may not be as severe as the complete loss-of-function induced in gene essentiality assays in the laboratory. For instance, human genes with mouse-essential orthologs were likely to be associated with the manifestation of severe and life-threatening diseases^38^. In addition, non-coding RNAs (ncRNAs), which often target and regulate the expression of hub proteins in PPI networks^39^, exhibited profound relevance to various biological pathways and diseases^40,41^, suggesting that they have rather tolerable implications on EGs. With respect to microRNA-mediated diseases, we observed that *w*-dependent EGs were associated with more dissimilar diseases than *k*-dependent EGs, although the numbers of associated diseases were not significantly different (Fig. S18). Therefore, with the growing resources for investigating ncRNAs and their relevance to diseases^42–45^, we expect that explorations of the association between stratified EGs and ncRNAs will provide useful insights into the molecular etiology of diseases.

One might ask whether the topology measures used here are robust to incompleteness of the networks. To answer that question, we investigated the correlations between *k*, *w*, and *f*_E_ in 100 random networks with 50% of the links removed. We found that both *k* and *w* were robust to the random changes of links (Fig. S19). With the removal of links, the correlations of *f*_E_ with *k* and *w* were only slightly decreased, and the difference was insignificant in most networks. In addition, the correlation between *k* and *w* remained close to 0, indicating that *k* and *w* would characterize distinct subsets of EGs. Therefore, we expect that our results will remain robust in more complete networks in the future.

Although our goal was to categorize different topology measures, one might also integrate various measures for the classification of EGs from non-EGs. Indeed, we found that the combination of centrality and clustering measures improved the power to predict EGs (see the *SI*; Fig. S20), although we simply used the rank of the topology measures as the predictive parameters. That strongly suggests that the application of more complicated statistical models or machine learning algorithms will further improve the prediction of EGs. In particular, recent studies have demonstrated that deep learning is a powerful approach to model complex genotype-phenotype associations^46,47^, providing insights into gene-disease associations^48^ and polypharmacy side-effects^49^. We believe that the incorporation of stratified topology measures with deep learning will improve the prediction of gene essentiality.

Regarding a gene as the unit of evolution, a gene might be selfish^50^ and establish a large number of clustered links with strong functional dependency (“strong links”), rendering itself indispensable for many cellular functions and thus ensuring its persistence in the population. In fact, many previous studies suggested a similar interpretation: that, rather than being crucial for global integration, central EGs simply have a greater chance to be involved in essential functions^13,17,20^. We observed that such selfishness is constrained, however; central EGs seemed to have weaker links than non-central EGs (Fig. 3B). From a systems perspective, a gene’s selfishness would not always be tolerable, as it comes at a fitness costs to the population. Assuming a system with such selfish genes of promiscuous functional relevance, a random failure may not be properly insulated, and the system would not be resilient to the frequent random errors in non-central nodes. This systems perspective asserts that strong links are constrained from connecting central nodes to other nodes. Indeed, strong links were found to be likely confined within local regions in various real networks including PPI networks^51,52^, genetic interaction networks^53^, brain connectomes^54^, and social networks^28^. That suggests that gene essentiality evolves in a tradeoff between a gene’s importance and its implication on system robustness, and one needs to synthesize gene-centric and system-centric perspectives for a comprehensive understanding of gene essentiality.

## Methods

### Relationships between gene essentiality and topology measures

The “consolidated” PPI networks were downloaded from the web interface to the Interaction Reference Index repository (iRefWeb)^55^ on June 7, 2017. The “binary” and “co-complex” networks were downloaded from the high-quality interactomes (HINT) database^56^ on June 27, 2017. The “high-quality” networks were created by combining the binary and co-complex networks. Gene essentiality information was downloaded from the online gene essentiality (OGEE) database^57^ on June 9, 2017. Any essentiality annotation with the “TextMining” data type was removed.

To explore a parameter’s ability to characterize gene essentiality, we calculated the Pearson correlation coefficient (*R*) between the fraction of EGs (*f*_E_) and the average of a given parameter along with the rank-ordered groups. Proteins were sorted by increasing order of the parameter of interest and added into a single bin until the bin contained at least 2% of the total population. This applied to all relationships of *f*_E_ with centrality measures (*DC*, *BC*, *CC*, and *EC*) and clustering measures (*C*, *μCXC*, *μLCC*, *μECC*, *ΣCXC*, *ΣLCC*, and *ΣECC*). See Table S7 in the *SI* for the definitions of topology measures.

For the PCA of EGs, we used the decomposition.PCA() object in the Python “scikit-learn” package. To scale the features, we also used the transform() function of the preprocessing.StandardScaler() object in the same package.

### Monitoring global and local connectivity upon pruning

Pruning analysis was performed in a manner similar to that previously reported^29^. Proteins were progressively removed from a given network at 5% of the total protein population in decreasing order of *k* and *w* while corresponding changes in Δ*C* with varying *f* (the fraction of removed proteins) were monitored. To calculate the Δ*C* of individual nodes, we constructed 100 random networks by degree sequence-preserved randomization^29,58^ and subtracted the mean node clustering coefficients of random sets from the observed node clustering coefficient.

To summarize the results of the pruning analyses, we measured the area between a given curve and the random curve. Because the decrease in Δ*C* was our interest, we specifically measured the area under the random curve. Therefore, if the given curve was over the random curve, the measured area became negative. We linearly interpolated the curves and calculated the trapezoidal area over *f* = [0, 0.95].

### Classification of *k*-dependent and *w*-dependent EGs

A gene could only be classified one of two ways: essential or non-essential. We assigned values of 1 to EGs and 0 to non-EGs. The probability that a given gene is essential was then calculated using logistic regression analysis according to a leave-one-out scheme, with *k* and *w* as dependent variables, resulting in *P*_E_(*k*) and *P*_E_(*w*), respectively. We performed the logistic regression analysis using the Python “scikit-learn” package. In addition, k_c_ (w_c_) was determined to maximize MCC regarding all nodes with *k* ≥ k_c_ (*w* ≥ w_c_) as predictive positives.

### Functional association between *k*-dependent and *w*-dependent EGs

To construct functional networks, we defined GO terms as *k*-functions if they were enriched with *k-*dependent genes in at least three PPI networks (*P* < 0.05, hypergeometric test); we also defined *w*-functions accordingly. GO terms were discarded when the number of genes annotated to them was less than three. Note that a GO term could be enriched with both *k*-dependent and *w*-dependent EGs, as the two types of enrichment were tested independently. A link was established between two GO terms if there was significant gene overlap (*P* < 10^−5^) between them. We used the MCODE application^59^ to identify clusters in the functional networks. For each cluster, we selected the median-sized GO term as the cluster’s representative function, where size is the number of genes annotated to the function. We constructed a total of six functional networks for three GO categories (BP, MF, and CC) and two eukaryotic species (yeast and human). Annotations were downloaded from the GO database^60,61^; the submission date of the human data used in the study was September 26, 2017, and that of yeast data was September 13, 2017.

### Impact of node removal on community structure

For each PPI network, we constructed a hierarchical organization based on the *Walktrap* algorithm^62^, using the Python package “python-igraph”. We chose the algorithm for its concept underlying the similarity between nodes. The algorithm relies on random walks to measure the similarity between two nodes by comparing their probability of random visits on other nodes: if two nodes are in the same community, then random walks starting from each node will visit all the other nodes in the same way. This process is somewhat reminiscent of the failure cascade shown in recent works, in which a single node failure was propagated and resulted in system-wide catastrophe^63^. After the similarity between nodes is established, the clustering process is agglomerative. In the earlier steps, nodes with greater similarity are put together into a community. Therefore, we took the fraction of prior merge steps, *f*_H_, as an indication of hierarchy; the smaller the *f*_H_, the lower the hierarchical level. By increasing *f*_H_ by 0.1 in a step-wise manner, we collected communities at different levels in the hierarchical organization. Communities comprising less than three members were discarded. The change in link density in community *s* upon the deletion of node *i* was calculated as follows: Δ*D*_*s,i*_ = (*l*_*s*_ − *l*_*s*,Δ*i*_)/(*n*_*s*_ × (*n*_s_ − 1)/2), where *l*_*s*_ denotes the number of links within *s* (i.e., two end nodes are both members of *s*), *l*_*s*,Δ*i*_ represents the number of links within *s* after removing node *i*, and *n*_*s*_ is the number of members in *s*. Therefore, Δ*D* measures the proportion of links removed upon a node deletion, indicating the extent of functional dependency within a community relying on the deleted node.

### Essential genes across contexts

For human cancer cell lines, we used a CRISPR screen dataset including 436 cell lines (gene_effect.csv, 18Q2) from DepMap database^64^. The expression level of genes was downloaded from the CCLE database^65^ with the matching version.

The essentiality of mouse genes was downloaded from the OGEE database^57^, similarly to that of human genes. Orthologs between human and mouse were identified from the Inparanoid database^66^ (version 8.0). Gene ages were downloaded from the ProteinHistorian database^67^; specifically, we used protein families predicted from the OrthoMCL and PANTHER databases and reconstructed ancestral history by asymmetric Wagner parsimony. We used the pre-calculated set of dN/dS for yeast^68^ and human^69^, for which evolutionary rates were computed with several species and the average taken.

### Diseases associated with miRNAs

The relationships between genes and disease were constructed by connecting gene-miRNA and miRNA-disease associations. For gene-miRNA associations, we used miRTarBase,^70^ discarding pairs with only “weak” evidence. For miRNA-disease associations, we used two different databases, HMDD^71^ and MDGHI^45^. Because MDGHI is a predictive approach, we applied an arbitrary cutoff and discarded all pairs with score smaller than 0.01.

## Supplementary information

Supplementary Information

Supplementary Tables

## Data Availability

All data generated or analyzed during this study are provided in this published article and its *Supplementary Information* files, and at sbi.postech.ac.kr/w/WEG.
