# Supplementary material for: Link clustering explains non-central and contextually essential genes in protein interaction networks
Source: Sci Rep. 2019 Aug 12;9:11672. doi: 10.1038/s41598-019-48273-3 (PMC6690968; doi:10.1038/s41598-019-48273-3)
Supplement: Supplementary file 1 — Supplementary Information [file 41598_2019_48273_MOESM1_ESM.pdf]

Link clustering explains non-central and contextually essential genes in protein interaction networks

Inhae Kim[1], Heetak Lee[1], Kwanghwan Lee[1], Seong Kyu Han[1], Donghyo Kim, and Sanguk Kim[1,2]*

[1]Department of Life Sciences, and [2]School of Interdisciplinary Bioscience and Bioengineering, Pohang University of Science and Technology, Pohang 790-784, Korea

Supplementary information

Topology measures

The definitions of the topology measures in the study are shown in **Table S7**. We aimed to characterize non-central essential genes (EGs), which might be relevant to contextual essentiality. In particular, we were interested in link clustering, since recent reports have shown that the change in the essentiality of a gene tends to depend on the neighbors with which the gene has strong functional association.

We investigated three link clustering measures: *ECC*[1], *LCC*[2,3], and *CXC*[3] (see **Table S7**). All three measures were intended to estimate the extent of functional association between two connected nodes by gauging how densely neighbors are connected around the two nodes. Among the measures, a key difference is how they normalize the density of connections. *ECC* normalizes neighborhood overlap over only one node's degree, whereas the normalization of *LCC* and *CXC* involve both nodes' degrees. In our dataset, we observed that *LCC* and *CXC* provided better characterization of non-central EGs than *ECC*, indicating that it is crucial to account for both nodes to properly estimate the functional association between the two nodes.

In addition, two previous studies have shown that the sum of *ECC* has the ability to predict gene essentiality[4,5]. However, for our goal to find non-central EGs, the orthogonality to centrality should also be tested. The sum of any link clustering measure would have the capacity to turn into a centrality measure because of its inherent correlation with the number of interactions. In our dataset, we indeed observed that the sums of link clustering measures exhibited strong correlations with the centrality measures (**Figure 2B**). Therefore, aggregation of link clustering by summation does not seem to work for our goal to separate non-central EGs.

For the comparison, we used four frequently used centrality measures: *DC*, *BC*, *CC*, and *EC*[6].

PPI networks

Different PPI networks describe distinct ranges of functional dependency between proteins, and those describing weak dependency might limit our analyses. For instance, Yu *et al.* showed that binary networks consist largely of transient signaling interactions and inter-complex connections, which likely convey less functional dependency than the interactions of other networks[7]. Indeed, for the distinction of central and non-central EGs, binary networks often failed to behave as other networks with respect to network structure and biological characteristics (**Figures S9−17**). We confirmed those observations in two recent unbiased human interactomes, BioPlex 2.0[8] and HI-II-14[9], which were detected by affinity-purification followed by mass-spectrometry (AP-MS) and yeast-2-hybrid (Y2H) studies, respectively. In BioPlex 2.0, we observed that the link clustering measures μCXC and μLCC were correlated with gene essentiality (f_E) but not with degree (k) (**Figure S21**). By contrast, in HI-II-14, the link clustering measures were not correlated with f_E, indicating that they failed to characterize gene essentiality in that binary network.

Combining centrality and link clustering for the prediction of gene essentiality

Considering that two network properties, centrality and link clustering, characterize distinct subsets of EGs, one might expect the utilization of both properties to improve the prediction of gene essentiality compared with reliance on a single property. We assessed the performance of gene essentiality prediction for four centrality measures (DC, BC, CC, and EC), two link clustering measures (μCXC and μLCC), and their pairwise combinations. Specifically, we used the rank percentile of each measure and their sums as prediction scores, without any model-fitting or training parameters. As class imbalance was expected to be present (i.e., EGs are likely to be outnumbered by

non-EGs), we monitored the area under precision-recall curves (the recall-weighted average precision) as the performance measure.

We found that combinations of centrality and link clustering measures were more effective in the prediction of gene essentiality than combinations of two centrality measures or two link clustering measures (**Figure 20**). In the human consolidated network, for instance, the link clustering coefficient μLCC had only the fifth best performance out of the six tested measures. When μLCC was combined with different centrality measures ('$CC + \mu LCC$', '$EC + \mu LCC$', and '$DC + \mu LCC$'), however, it achieved the first, second, and third best performance out of 15 combinations of measures. By contrast, such improvement was not observed when the two link clustering measures were combined ('$\mu CXC + \mu LCC$'). That observation was robust in different PPI networks: in seven out of eight PPI networks, the combination of centrality measures and link clustering measures provided the best performance.

References

1. Radicchi, F., Castellano, C., Cecconi, F., Loreto, V. & Parisi, D. Defining and identifying communities in networks. *Proc. Natl. Acad. Sci. U. S. A.* **101**, 2658–63 (2004).
2. Onnela, J.-P. *et al.* Structure and tie strengths in mobile communication networks. *Proc. Natl. Acad. Sci. U. S. A.* **104**, 7332–6 (2007).
3. Pajevic, S. & Plenz, D. The organization of strong links in complex networks. *Nat. Phys.* **8**, 429–436 (2012).
4. Wang, J., Li, M., Wang, H. & Pan, Y. Identification of essential proteins based on edge clustering coefficient. *IEEE/ACM Trans. Comput. Biol. Bioinforma.* **9**, 1070–1080 (2012).
5. Li, M., Zhang, H., Wang, J. & Pan, Y. A new essential protein discovery method based on the integration of protein-protein interaction and gene expression data. *BMC Syst. Biol.* **6**, 15 (2012).
6. Newman, M. E. J. Mathematics of Networks. in *The New Palgrave Dictionary of Economics* **119**, 1–8 (Palgrave Macmillan UK, 2008).
7. Yu, H., Braun, P., Yıldırım, M. & Lemmens, I. High-quality binary protein interaction map of the yeast interactome network. *Science (80-.).* **104**, (2008).

8. Huttlin, E. L. *et al.* The BioPlex Network: A Systematic Exploration of the Human Interactome. *Cell* **162**, 425–440 (2015).

9. Rolland, T. *et al.* A Proteome-Scale Map of the Human Interactome Network. *Cell* **159**, 1212–1226 (2014).

Supplementary tables

Table S1−6 were provided as an excel file.

Table S7 | Topology measures in this study

Type		Name	Denotes	Definition	
Clustering	Node	Node clustering coefficient	*C*	$C_i = \frac{2T_i}{k_i(k_i - 1)}$	
	Link	Product of node clustering coefficient	*CXC*	$CXC_{ij} = (C_i + 1)(C_j + 1)$	if $A_{ij} = 1$
		Link clustering coefficient	*LCC*	$LCC_{ij} = \frac{n_{ij}}{[(k_i - 1) + (k_j - 1) - n_{ij}]}$	
		Edge clustering coefficient	*ECC*	$ECC_{ij} = \frac{n_{ij} + 1}{min[(k_i - 1), (k_j - 1)]}$	
	Aggregate of link measures	Average of CXC	*μCXC, w*	$\mu CXC_i = \frac{\sum_j CXC_{ij}}{k_i}$	
		Average of LCC	*μLCC*	$\mu LCC_i = \frac{\sum_j LCC_{ij}}{k_i}$	
		Average of ECC	*μECC*	$\mu ECC_i = \frac{\sum_j ECC_{ij}}{k_i}$	
		Sum of CXC	*ΣCXC*	$\Sigma CXC_i = \sum_j CXC_{ij}$	
		Sum of LCC	*ΣLCC*	$\Sigma LCC_i = \sum_j LCC_{ij}$	
		Sum of ECC	*ΣECC*	$\Sigma ECC_i = \sum_j ECC_{ij}$	
Centrality		Degree centrality	*DC, k*	$DC_i = \frac{\sum_j A_{ij}}{\lvert \mathbf{V} \rvert}$	
		Betweenness centrality	*BC*	$BC_i = \sum_{s \neq i \neq t \in \mathbf{V}} \frac{\sigma_{st}(i)}{\sigma_{st}}$	
		Closeness centrality	*CC*	$CC_i = \frac{\lvert \mathbf{V} \rvert - 1}{\sum_{t \in \mathbf{V}} d(i,t)}$	
		Eigenvector centrality	*EC*	$\lambda \mathbf{EC} = \mathbf{A} \cdot \mathbf{EC}, \mathbf{EC} = (EC_1, EC_2, \ldots)$	

A, the adjacency matrix, $A_{ij} = 1$ if two nodes *i* and *j* are connected, $A_{ij} = 0$ otherwise; **V**, the set of nodes in the network; T_i, the number of triangles through node *i*; n_{ij}, the number of common neighbors of nodes *i* and *j*; σ_{st}, the number of shortest paths between nodes *s* and *t*; $\sigma_{st}(i)$, σ_{st} through node *i*; $d(i,t)$, the length of the shortest path between nodes *i* and *t*

Supplementary figures

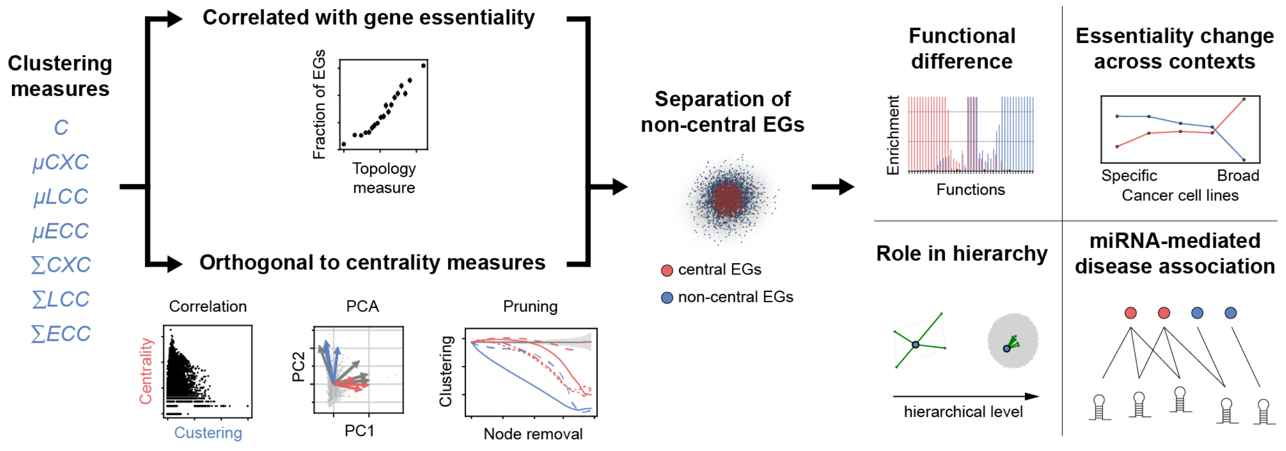

Figure S1. Flowchart of the manuscript. Clustering measures were tested for their capability to characterize gene essentiality and orthogonality to centrality measures. Relying on the selected clustering measure, EGs were classified as central or non-central. The two groups of EGs were compared for various biological aspects.

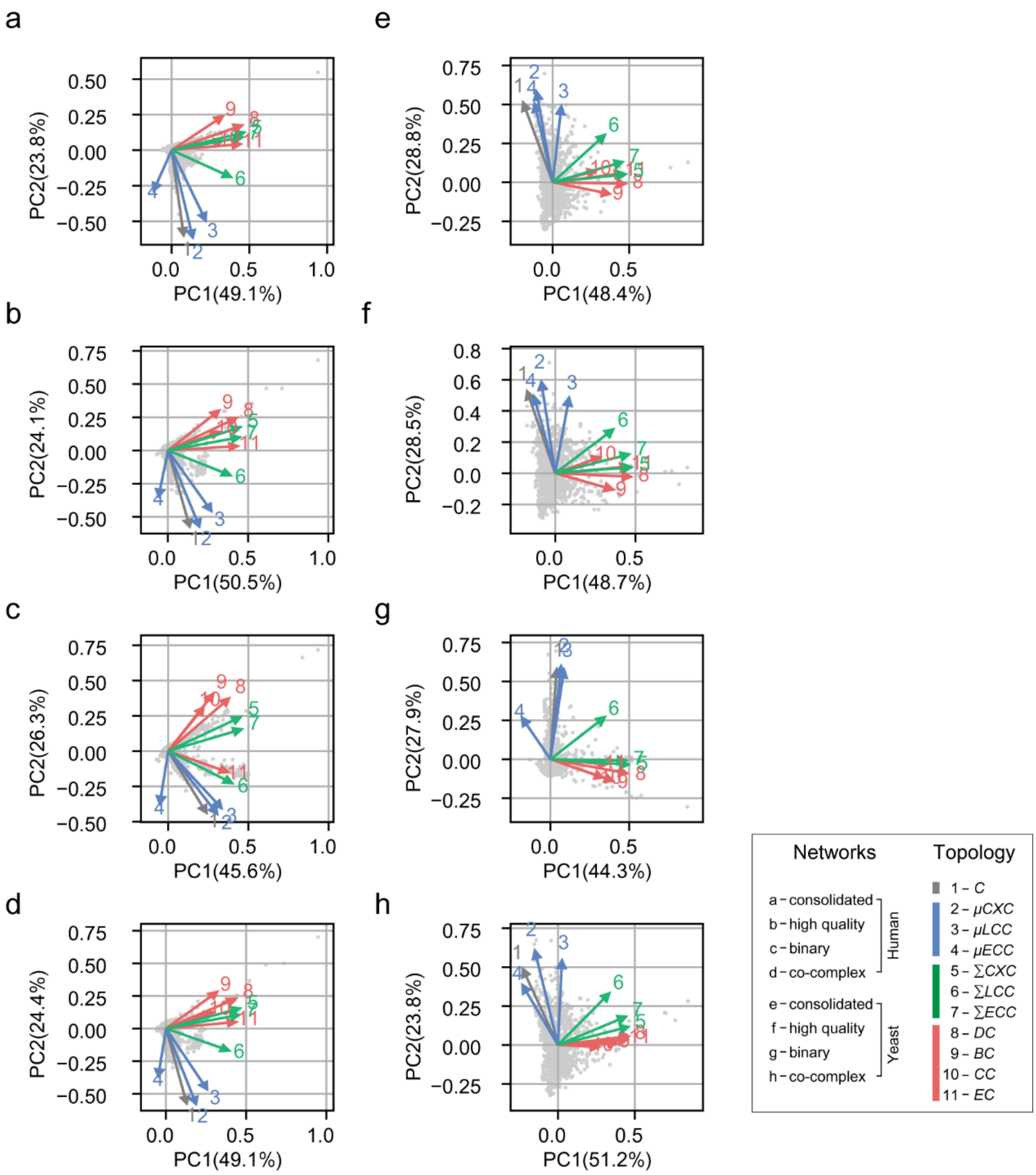

Figure S2. PCA analysis of topology measures on EGs in different PPI networks.

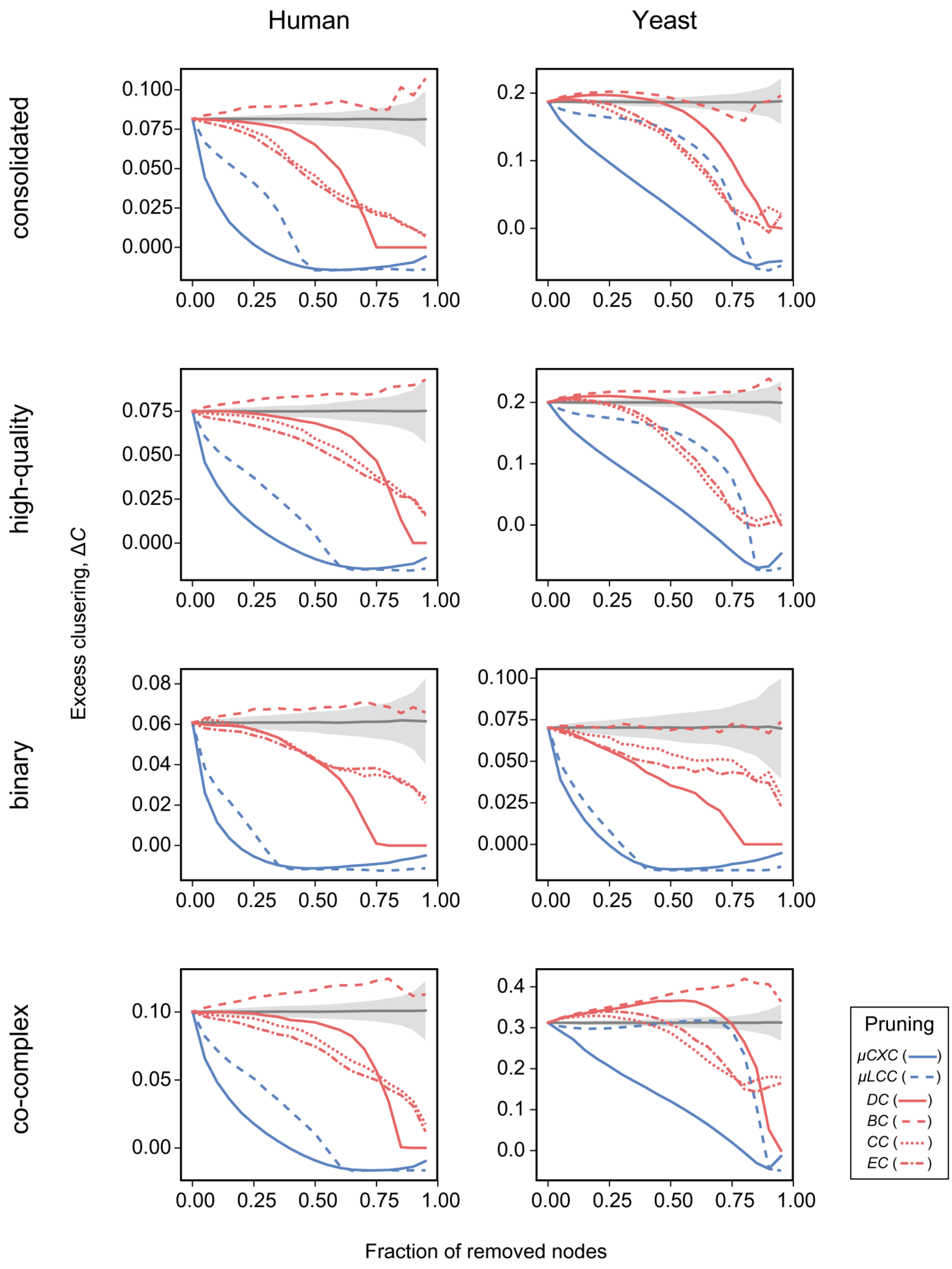

Figure S3. Pruning analyses in different PPI networks.

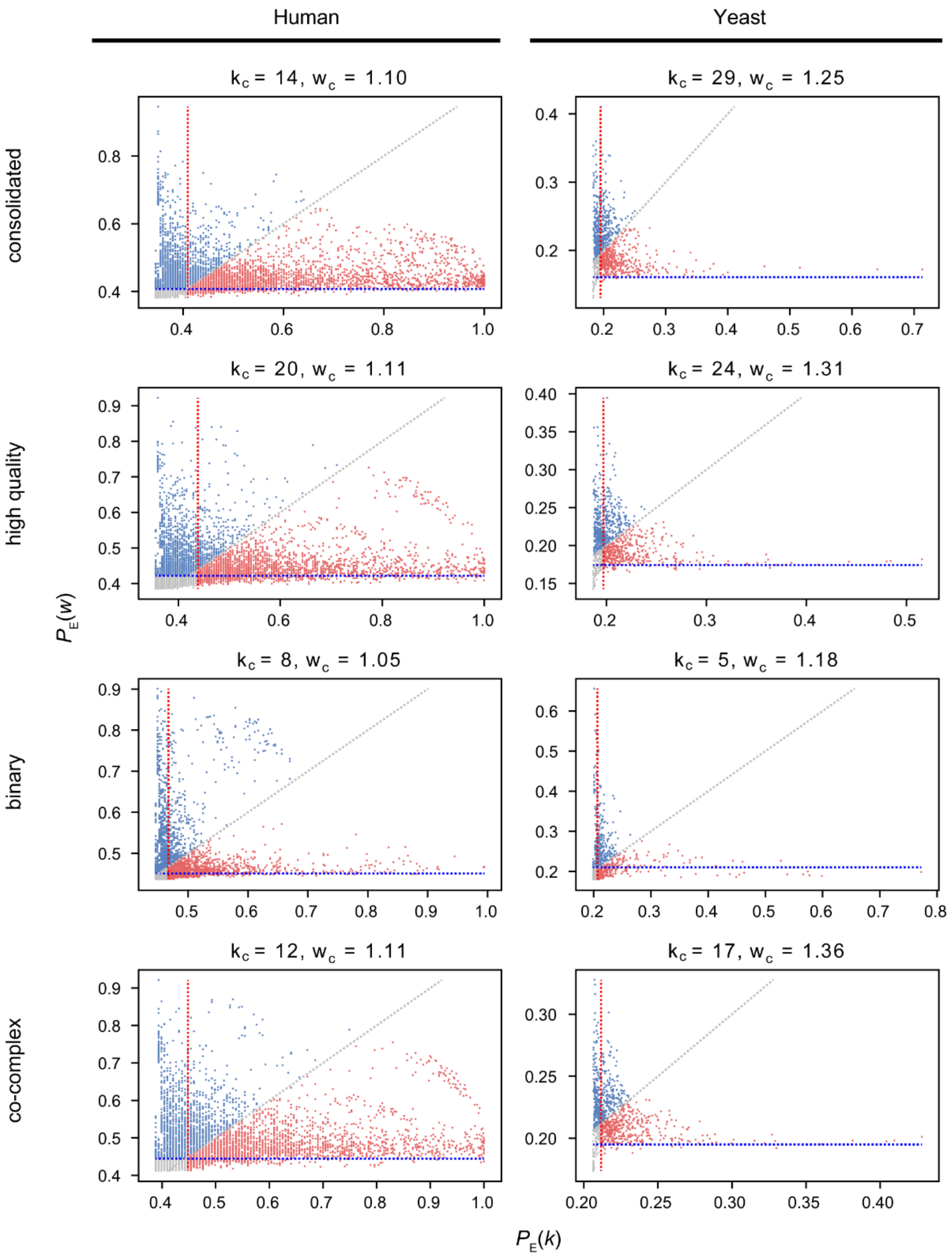

Figure S4. Probability of a gene being essential based on *k* and *w* for all EGs and the criteria to classify *k*-dependent and *w*-dependent EGs in different PPI networks. Criteria are shown as follows: gray dotted lines, $P_E(k) = P_E(w)$; red dotted lines, $k = k_c$; blue dotted lines, $w = w_c$.

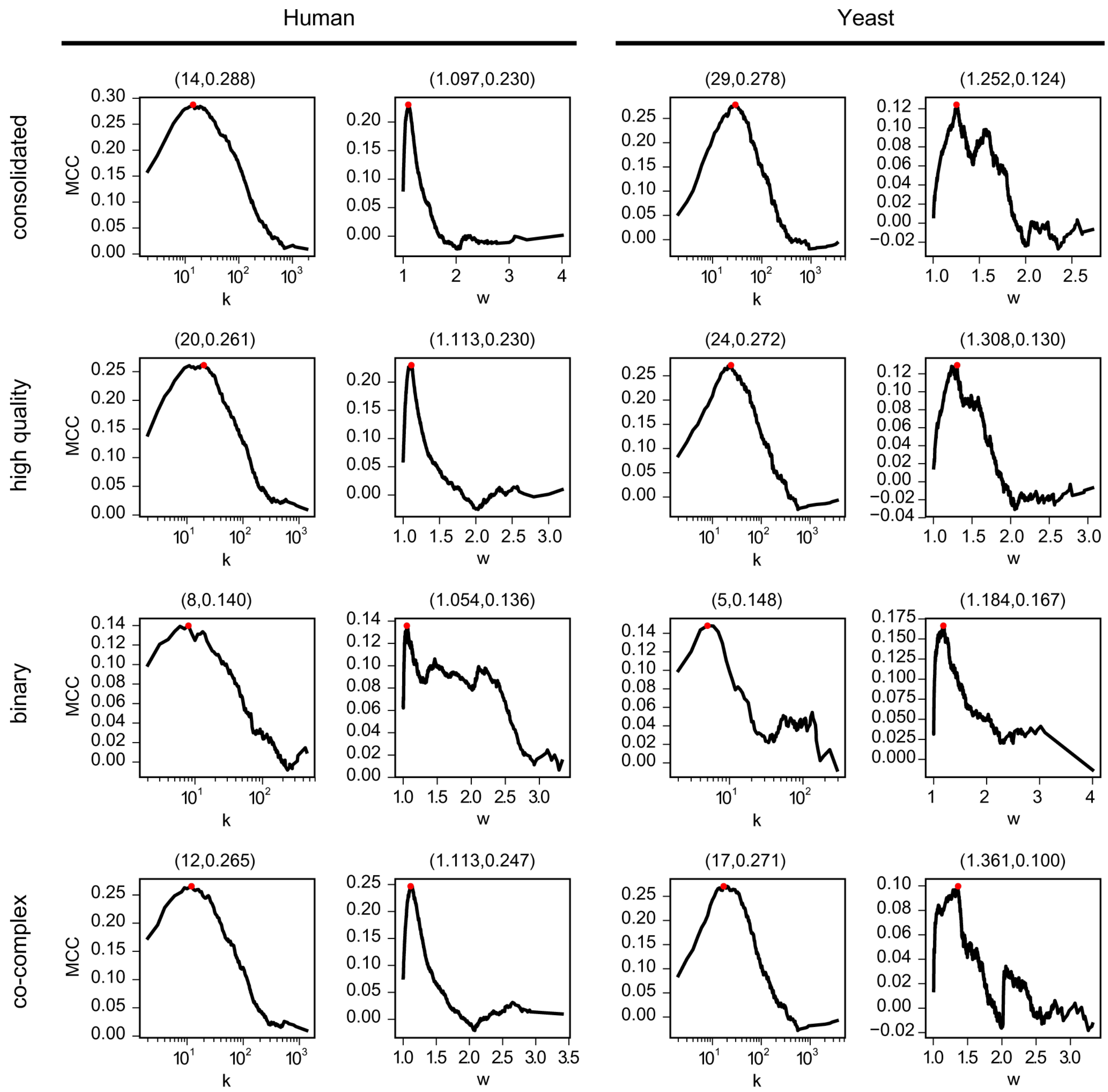

Figure S5. Finding the cutoffs, k_c and w_c, to maximize MCC. Red circles indicate points of maximum MCC. Numbers in parentheses indicate *k* or *w* with the maximum MCC.

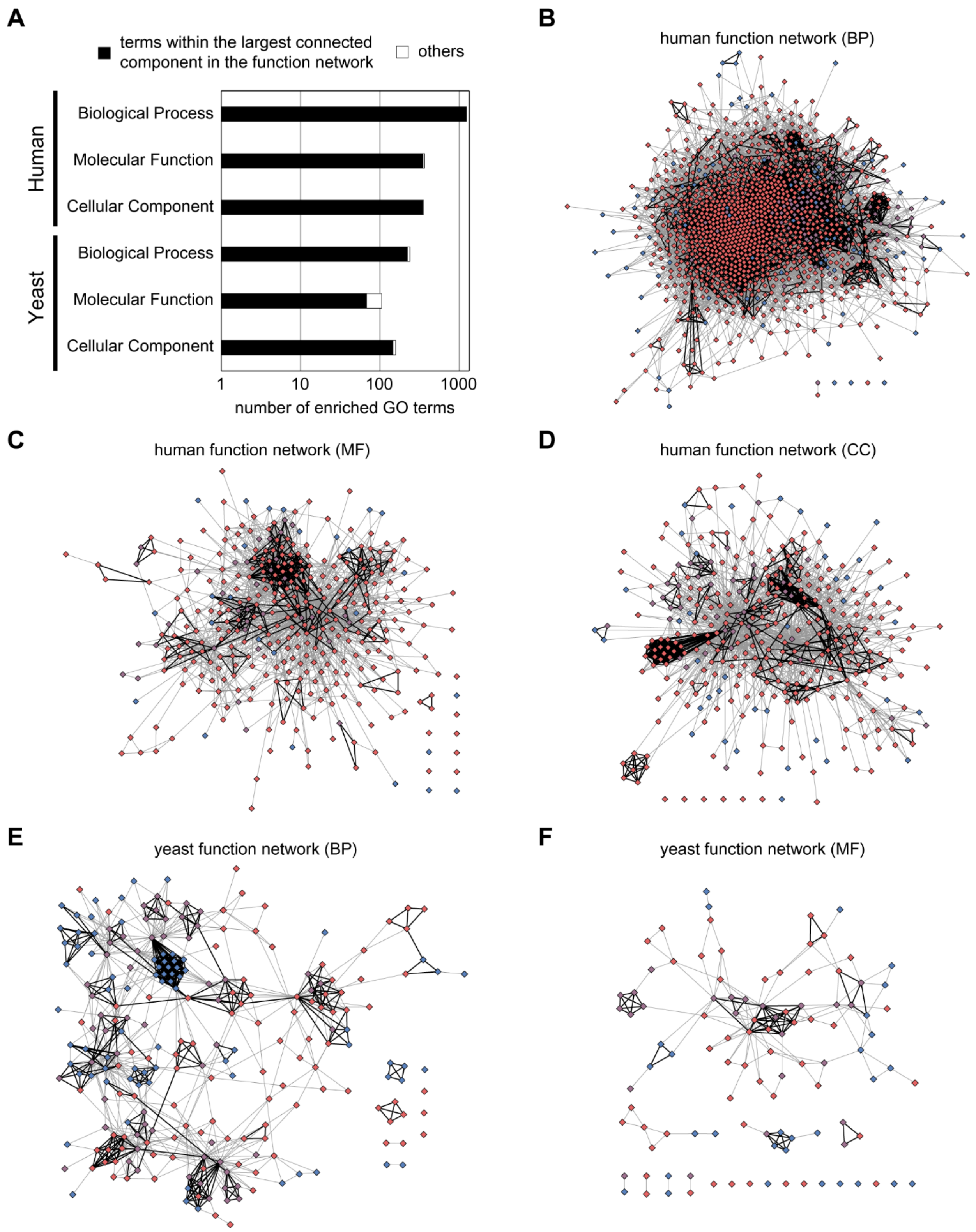

Figure S6. Functional networks for different GO categories. (A) The number of GO terms connected to the largest connected component in the functional network. (B−F) Visualization of other functional networks.

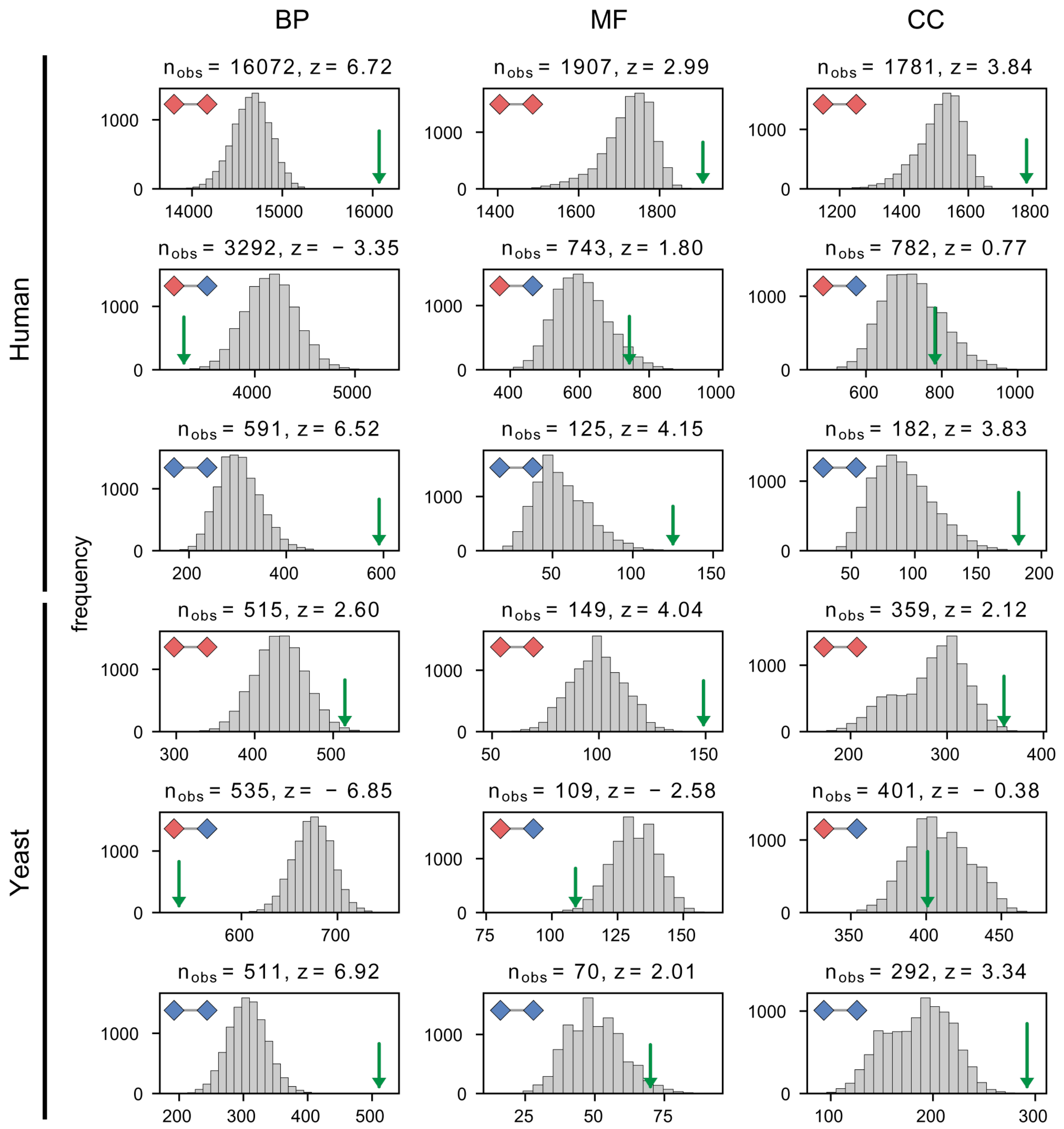

Figure S7. Number of links between *k*-functions and *w*-functions in real functional networks (green arrow) and in random sets (gray bars). Random sets were constructed by shuffling *k*-function and *w*-function tags 10,000 times.

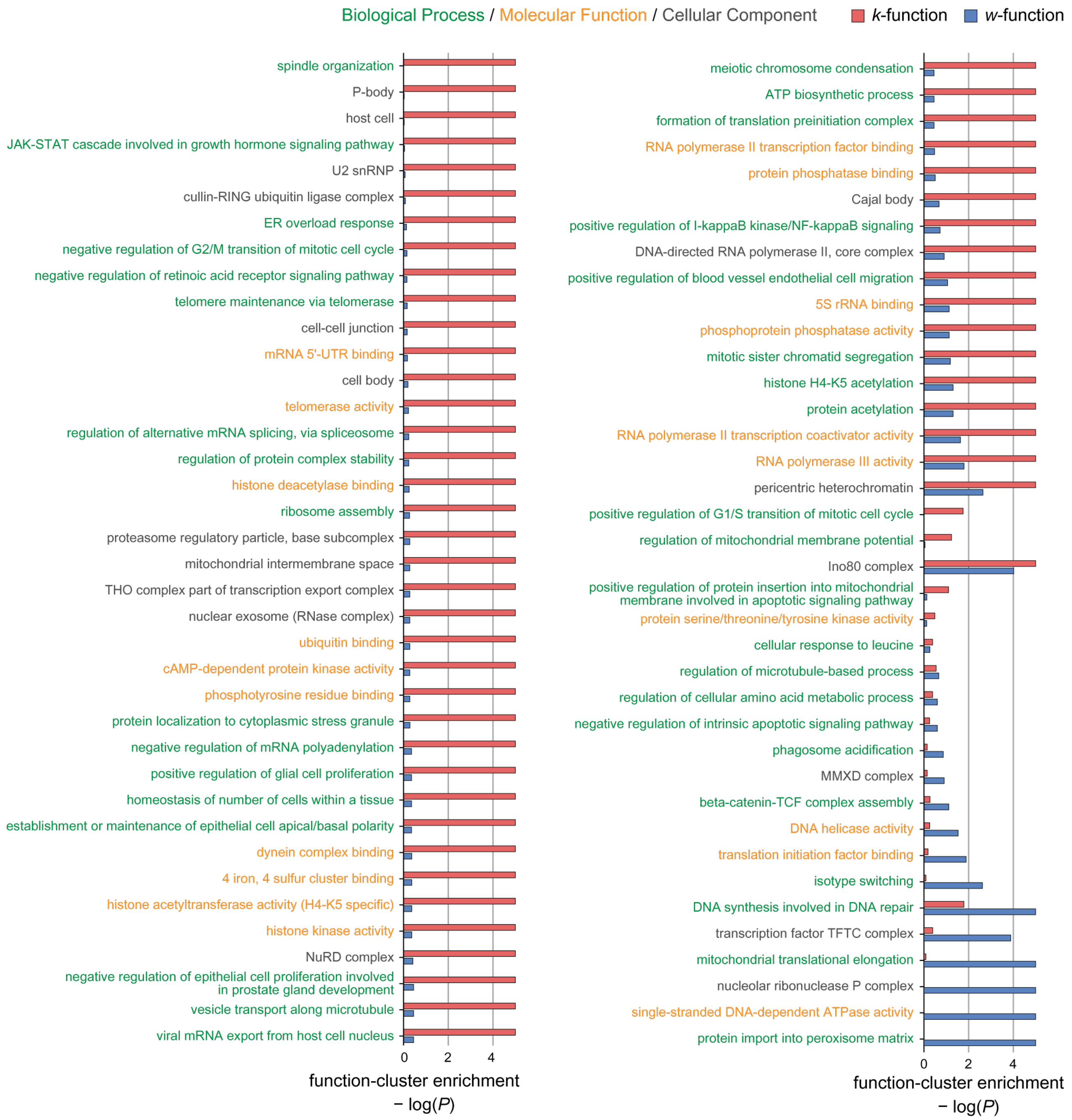

Figure S8. Representative GO terms of clusters from human functional networks and the bias of clusters toward *k*-functions and *w*-functions.

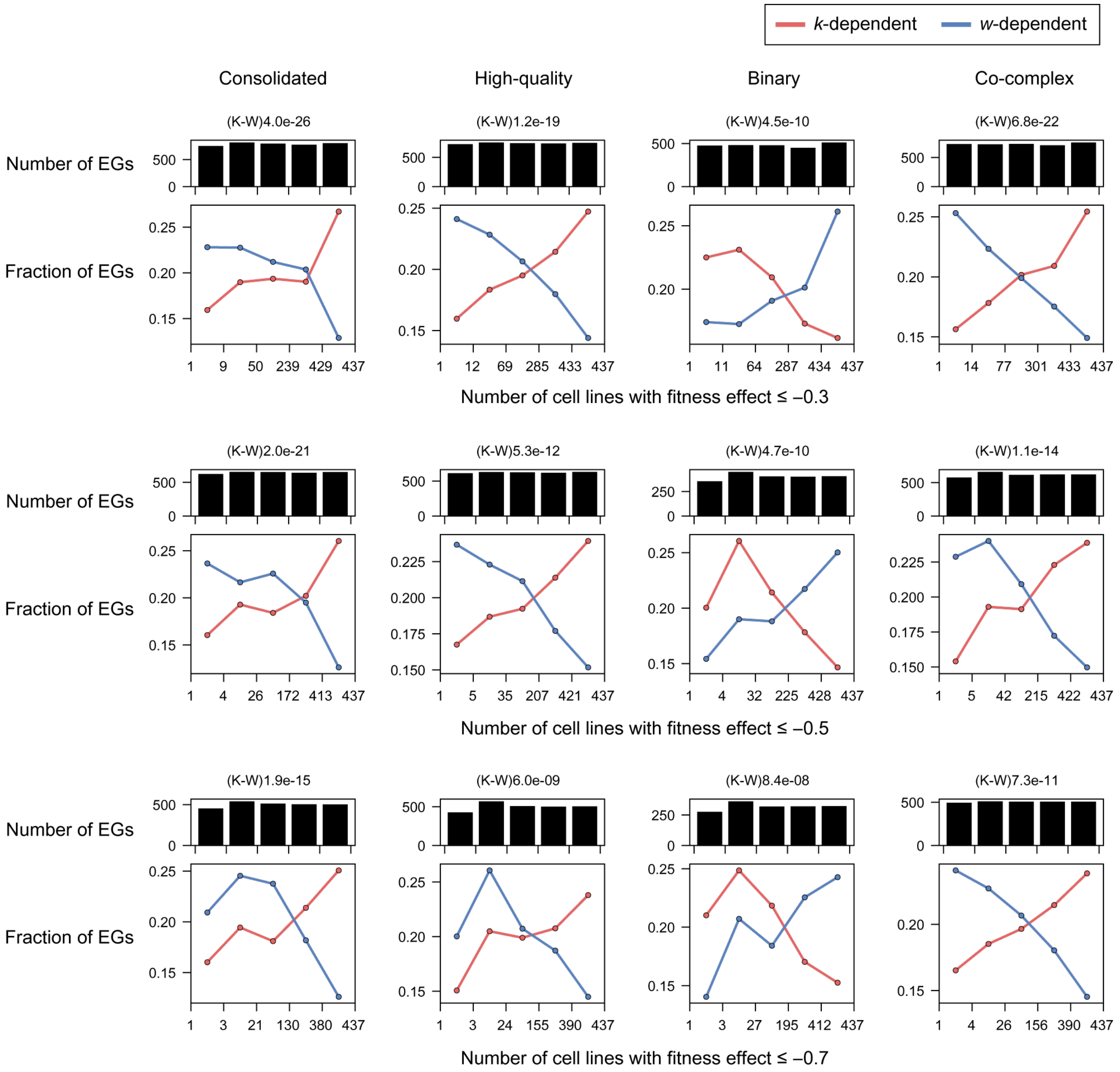

Figure S9. Contextual essentiality of *k*-dependent and *w*-dependent EGs with varying fitness effect cutoffs in different PPI networks.

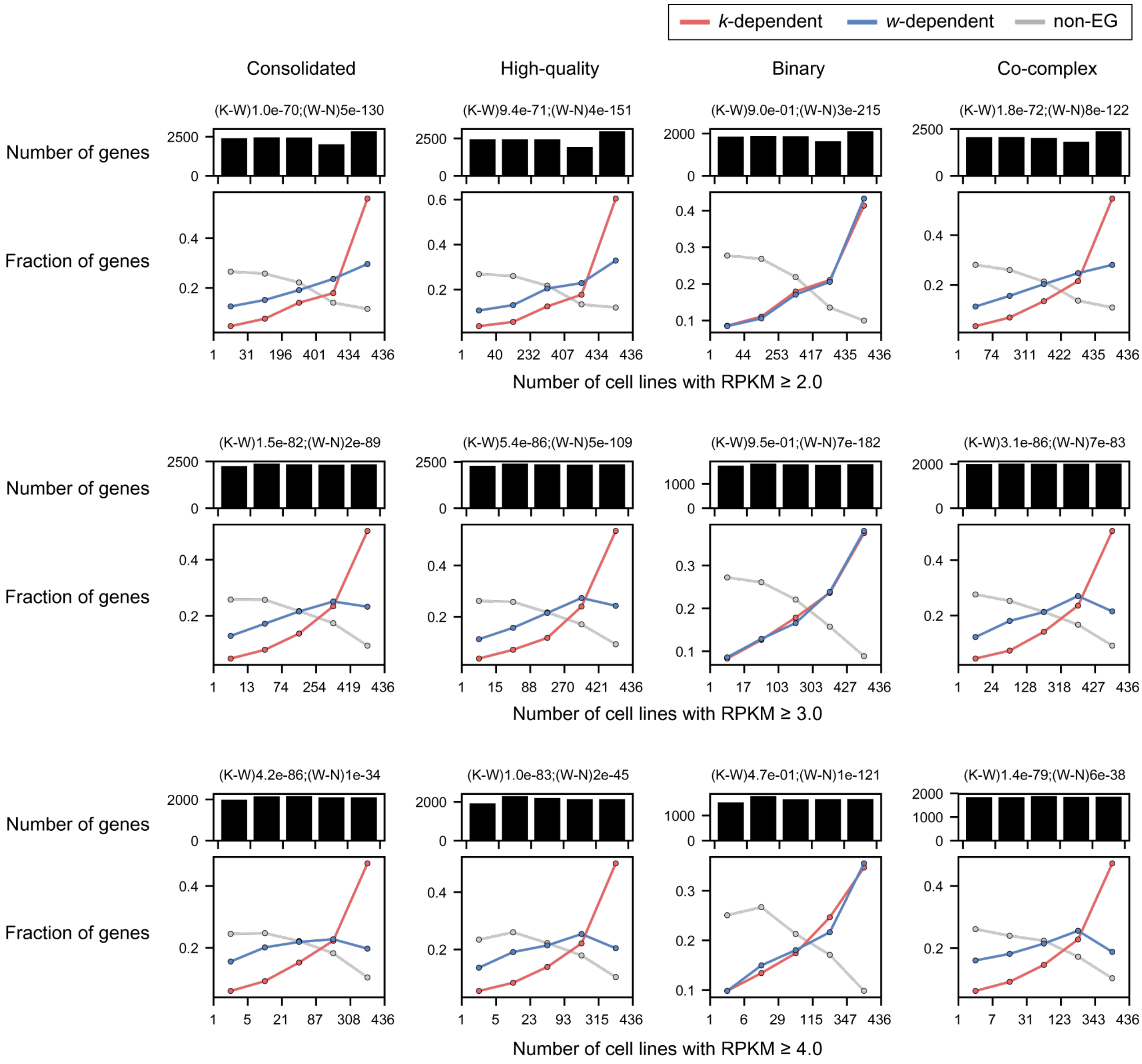

Figure S10. Contextual gene expression of *k*-dependent and *w*-dependent EGs in different PPI networks.

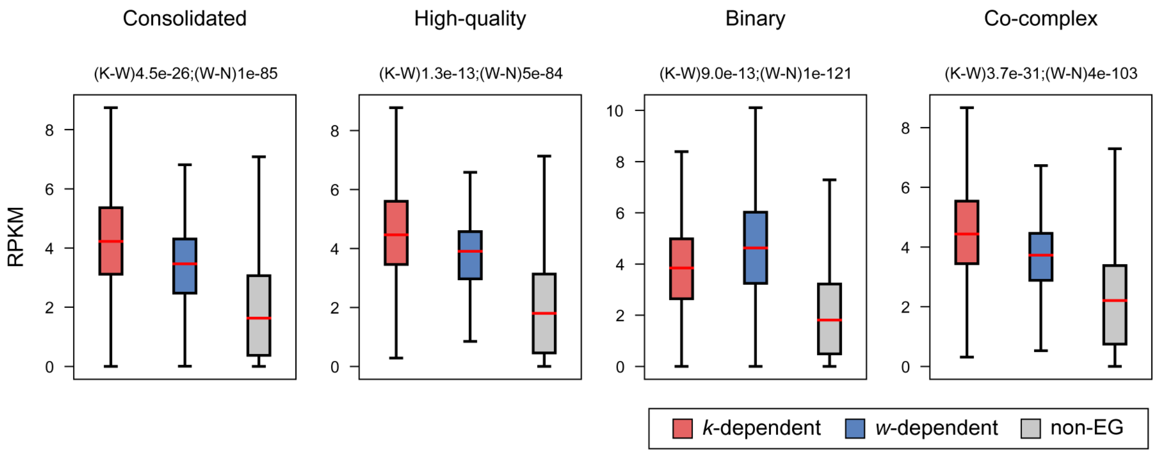

Figure S11. Average expression levels of *k*-dependent and *w*-dependent EGs and non-EGs.

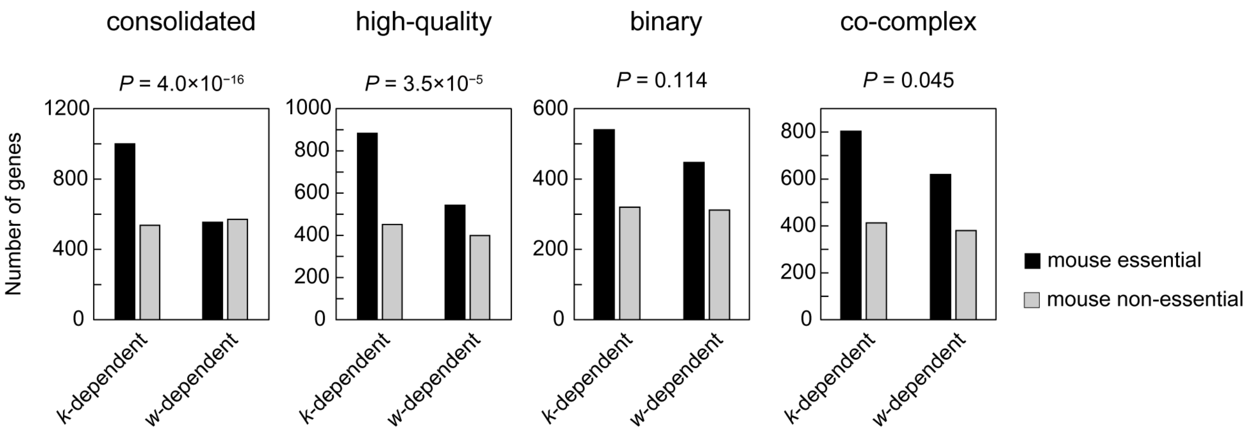

Figure S12. The essentiality of mouse orthologues of *k*-dependent and *w*-dependent EGs in different PPI networks.

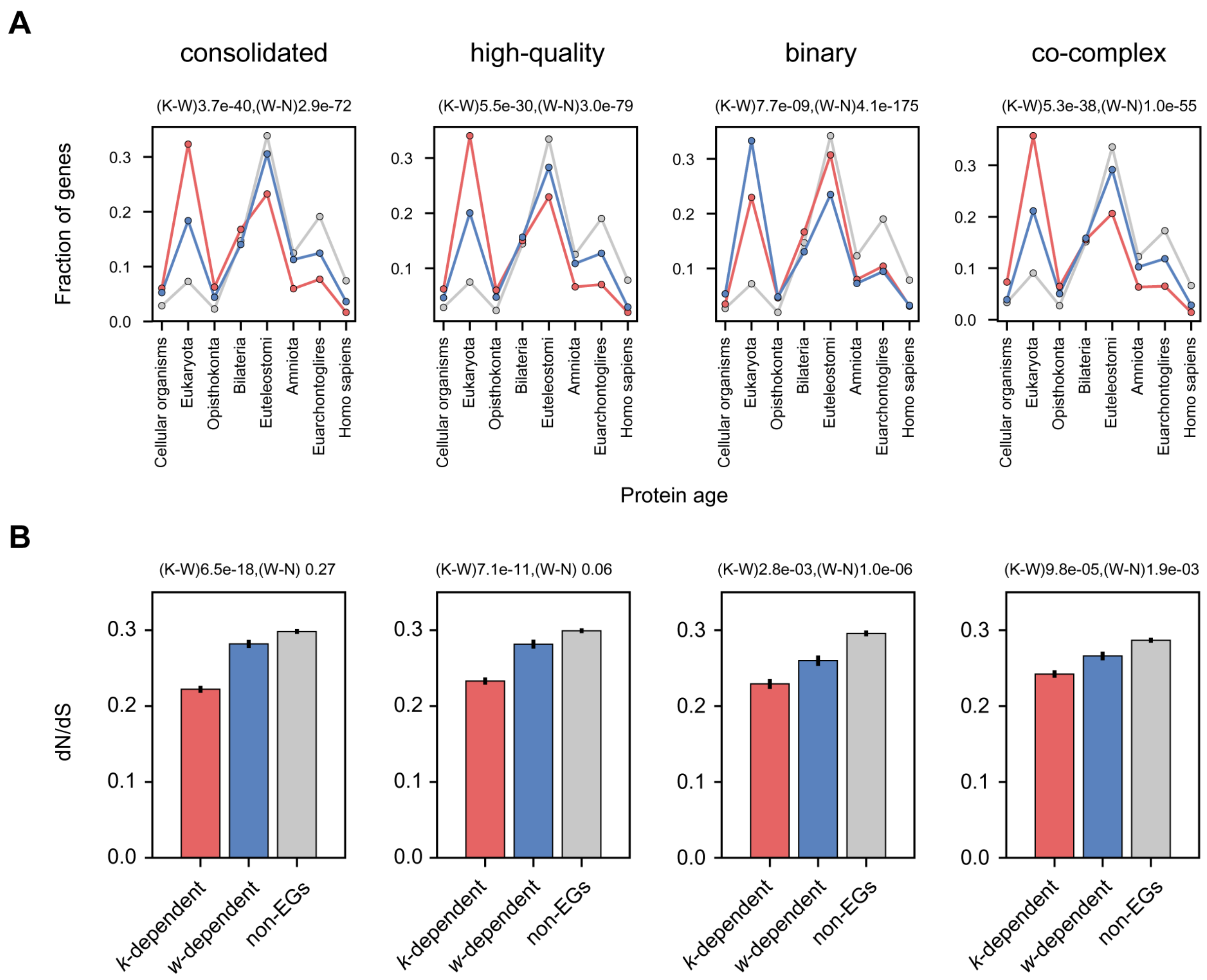

Figure S13. Evolutionary conservation of *k*-dependent and *w*-dependent EGs. (A) Fraction of genes distributed over phyletic ages. (B) Evolutionary rate (dN/dS) of genes (error bar = standard error).

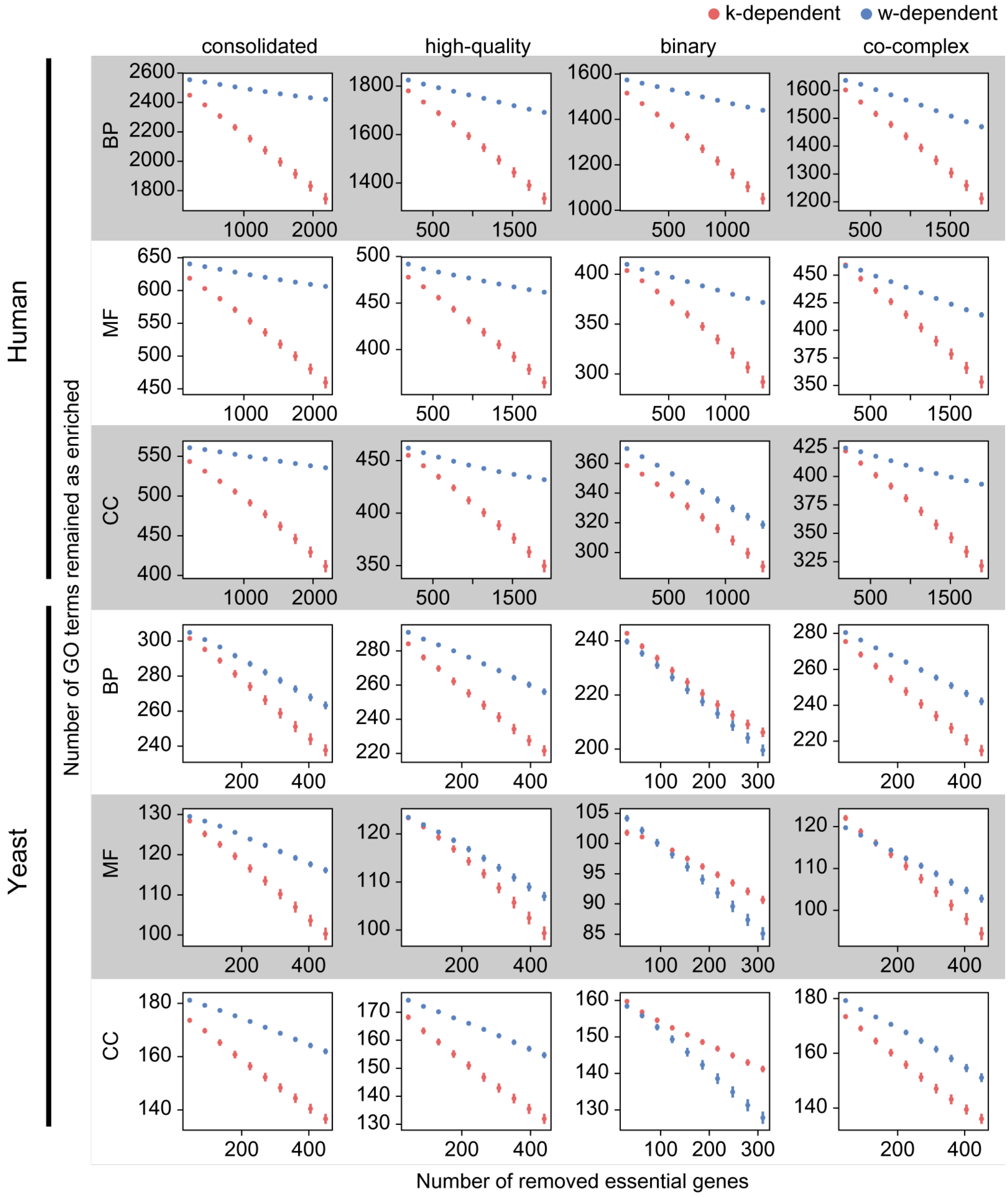

Figure S14. The implication of *k*-dependent and *w*-dependent EGs on the functional modules. The number of GO terms enriched with *k*-dependent or *w*-dependent EGs were monitored after removal of the same number of *k*-dependent and *w*-dependent EGs, which were randomly selected 100 times (error bars = standard deviation).

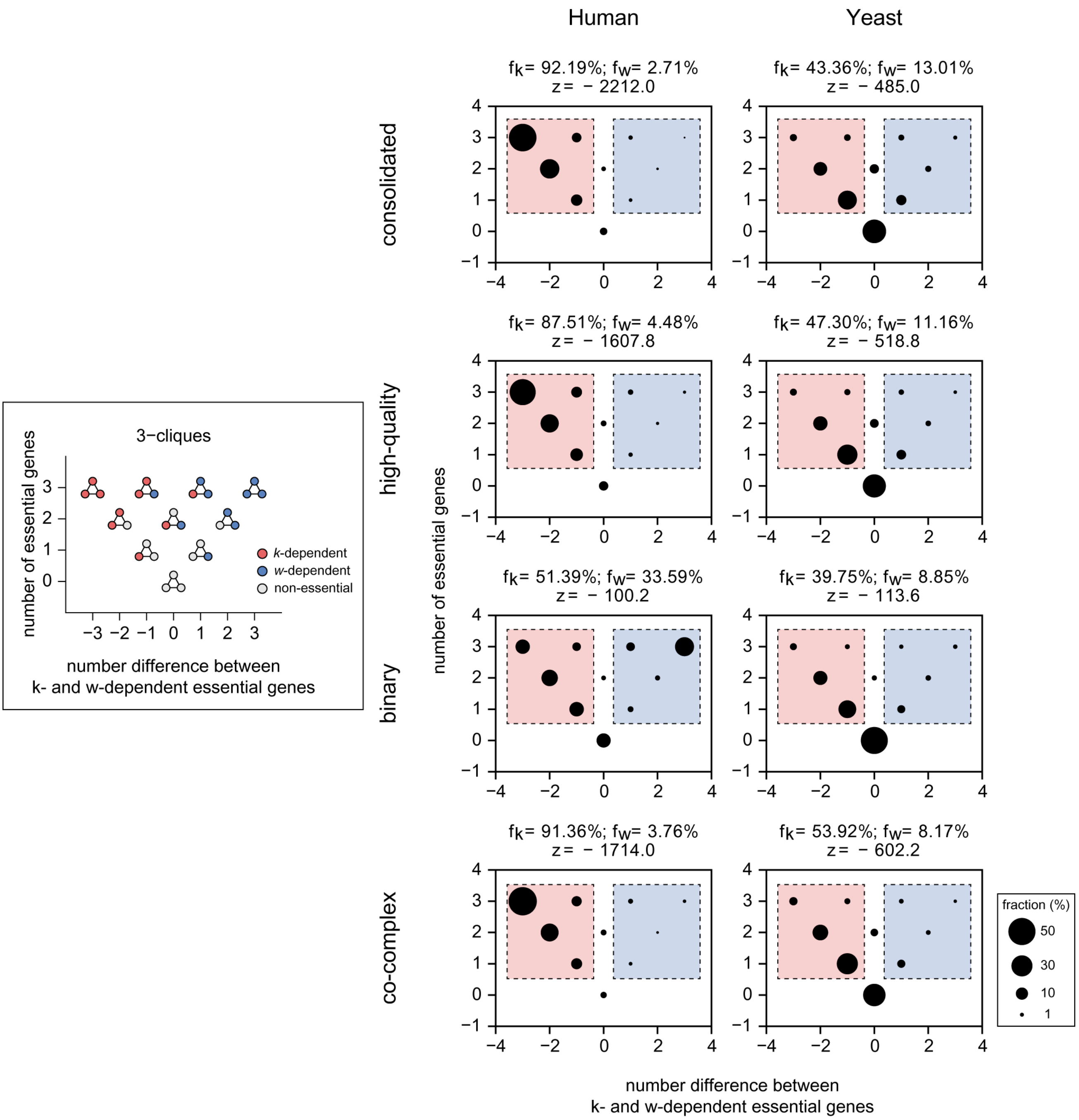

Figure S15. Biases in 3-cliques toward *k*-dependent and *w*-dependent EGs in different PPI networks.

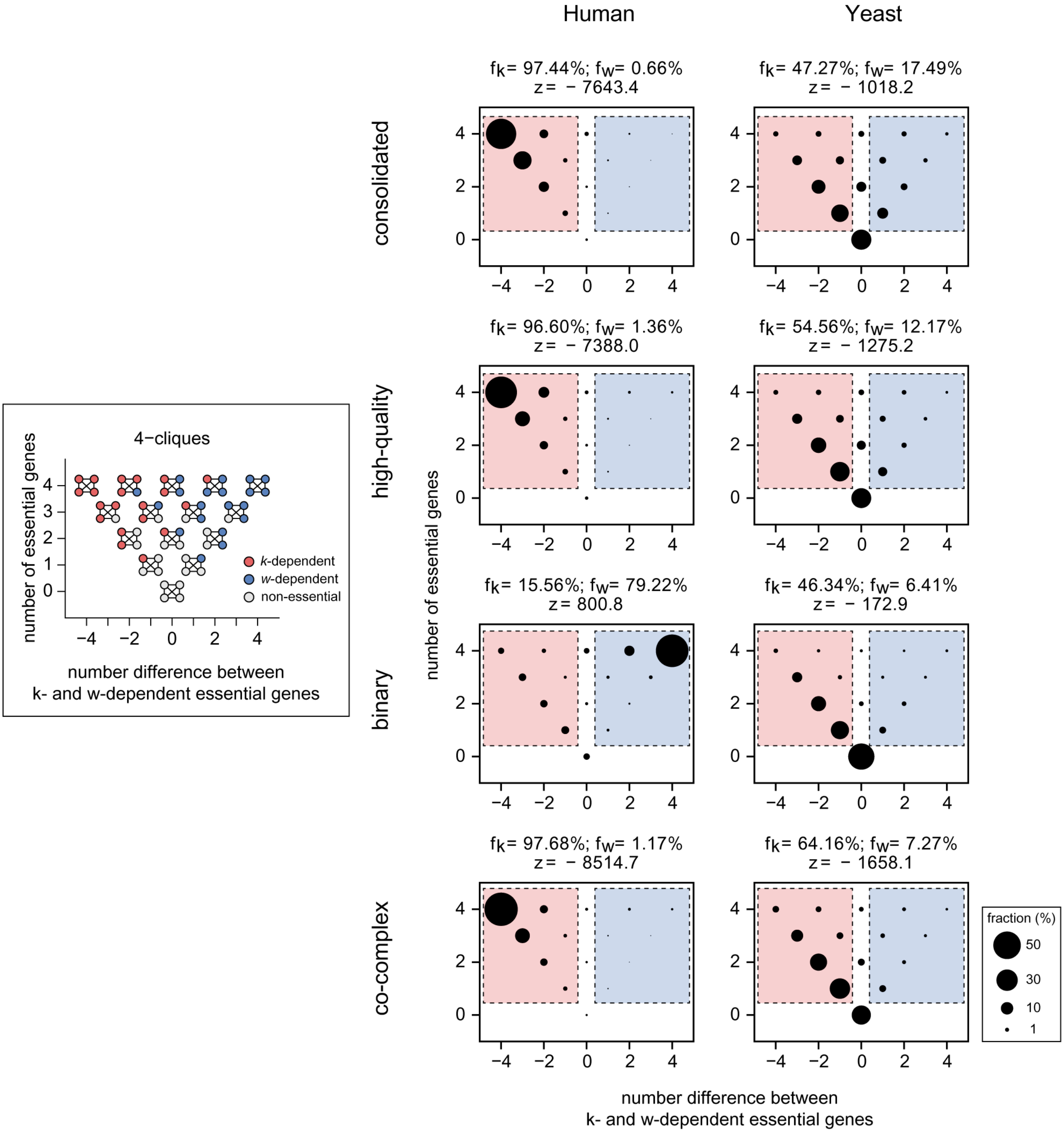

Figure S16. Biases in 4-cliques toward *k*-dependent and *w*-dependent EGs in different PPI networks.

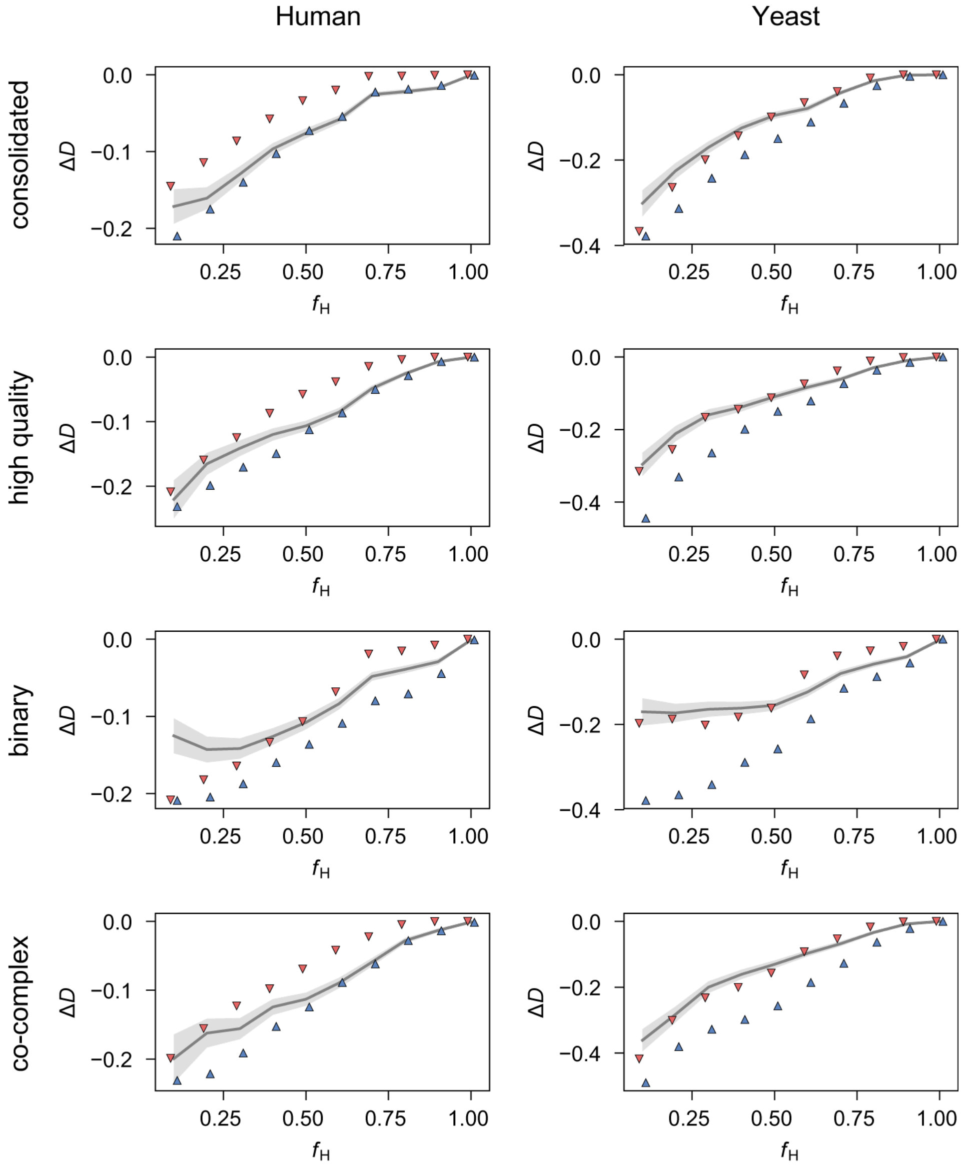

Figure S17. Change of the link density in the community structure upon removal of a single node (Δ*D*) at different levels of hierarchy in different PPI networks. The average change of link density for the removal of *k*-dependent and *w*-dependent EGs is shown as red downward and blue upward triangles, respectively. Gray lines indicate the averages for non-EGs (area = standard error).

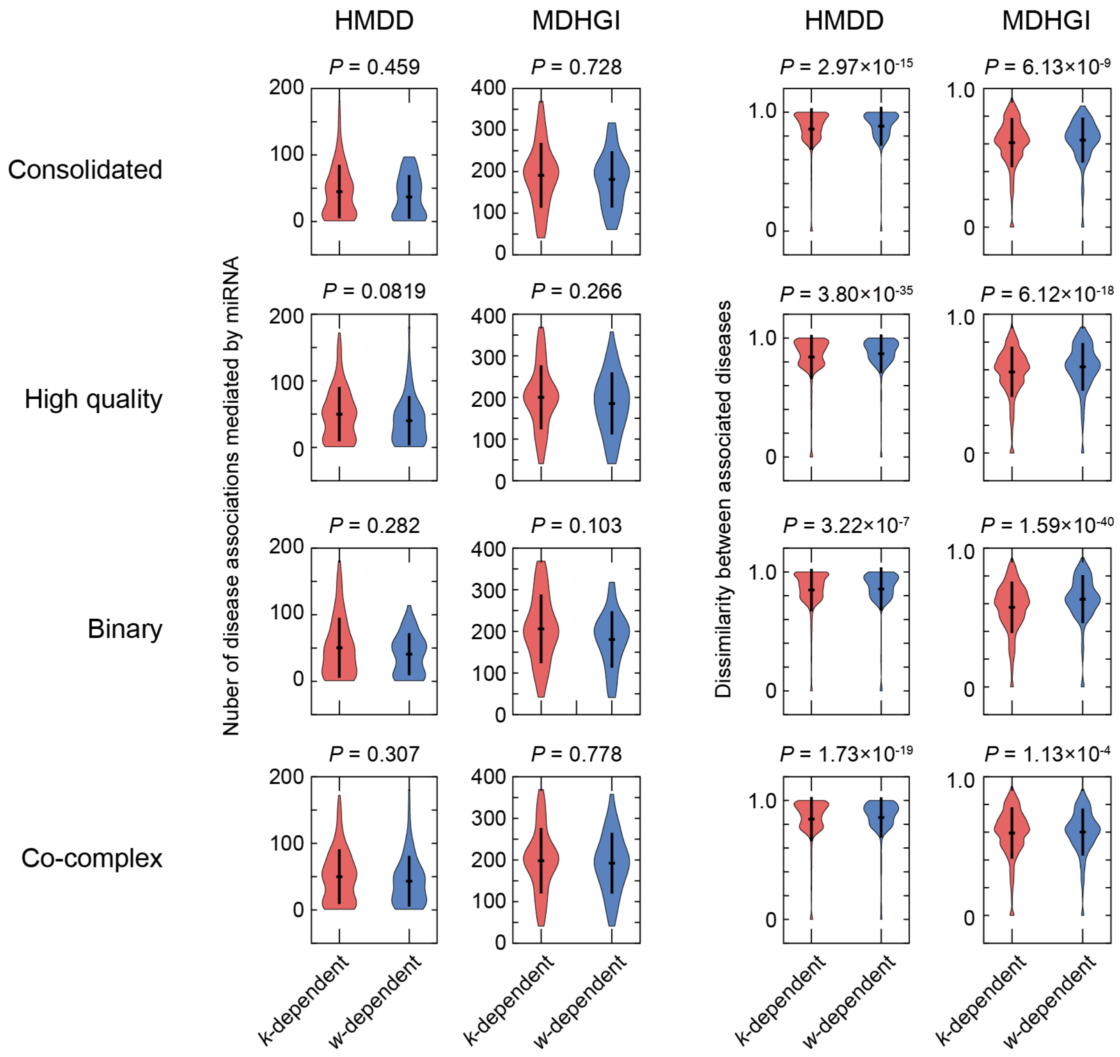

Figure S18. miRNA-mediated associations between EGs and diseases. (A) The number of associated diseases for *k*-dependent and *w*-dependent EGs. (B) Dissimilarity of associated disease for *k*-dependent and *w*-dependent EGs. Dissimilarity between two genes *i* and *j* was calculated as $1 - J(\mathbf{D}_i, \mathbf{D}_j)$, where *J* is the Jaccard index and $\mathbf{D}_i$ is the set of diseases associated with gene *i* through miRNAs.

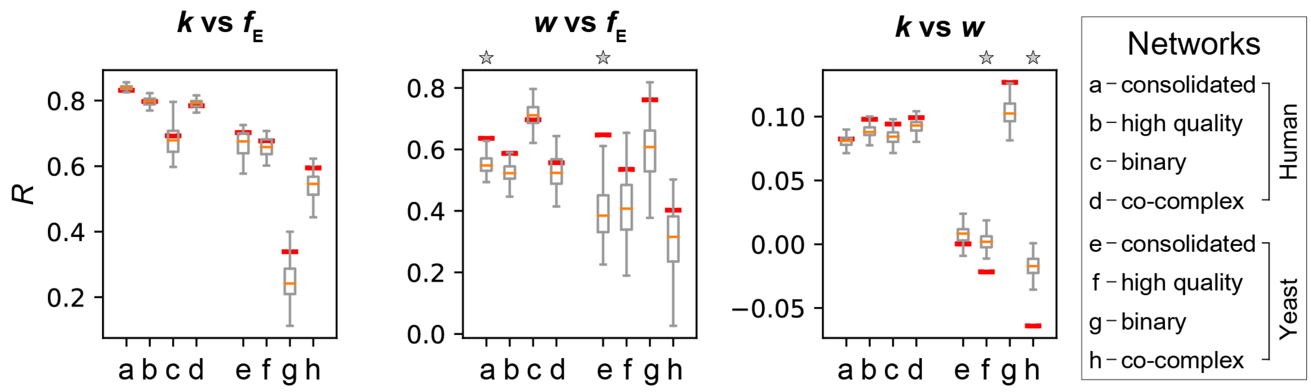

Figure S19. Correlations between k, w, and f_E with random removal of links. Pearson correlation coefficients (R) were calculated in 100 random networks with 50% of the links removed. The red horizontal line indicates the observed correlation in real networks (gray star, $|z| \geq 2.58$).

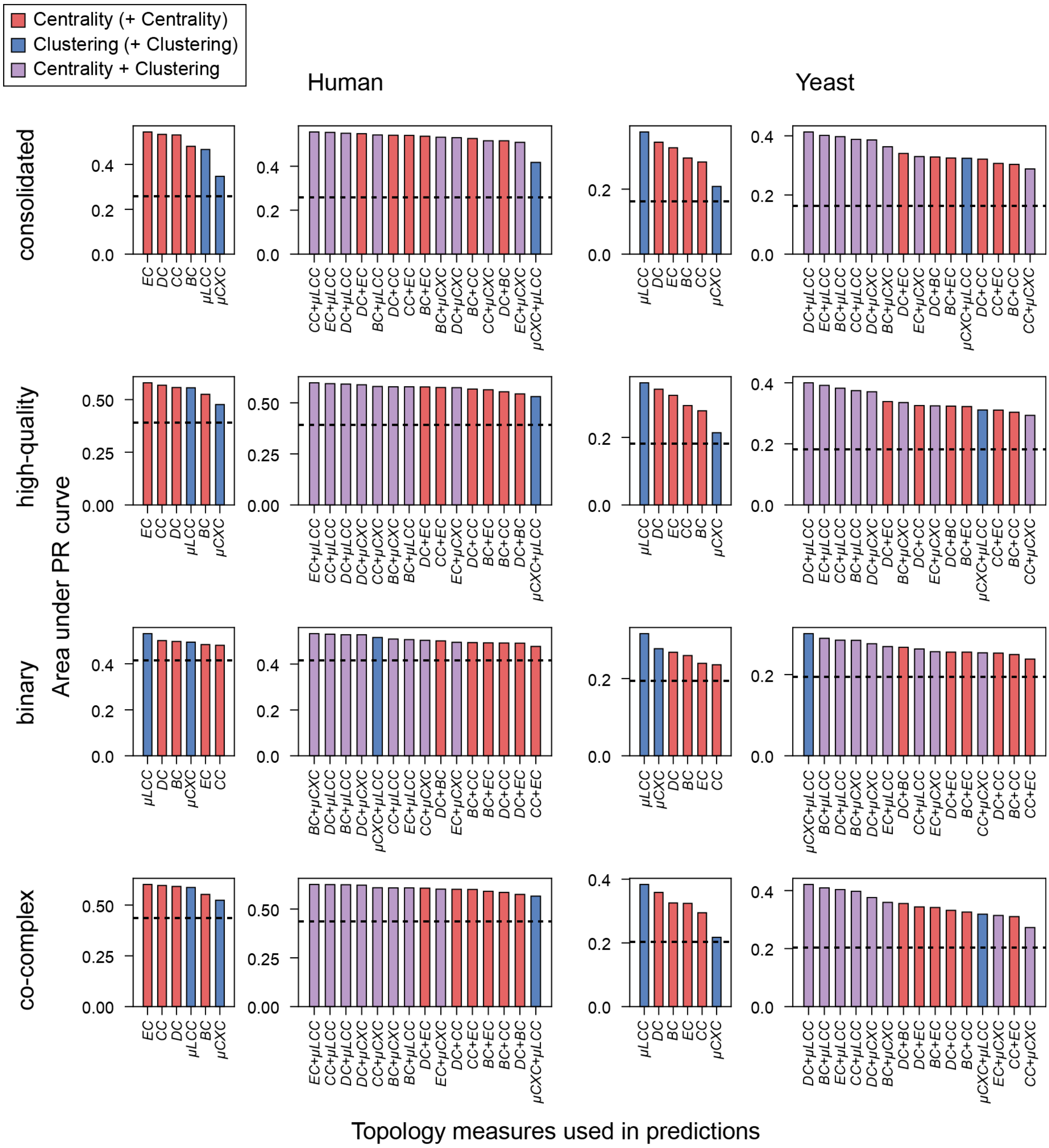

Figure S20. Performance of centrality measures and link clustering measures in eight different PPI networks. The performance was measured by the area under the precision-recall curve (AUPRC); the dotted line indicates the expected AUPRC.

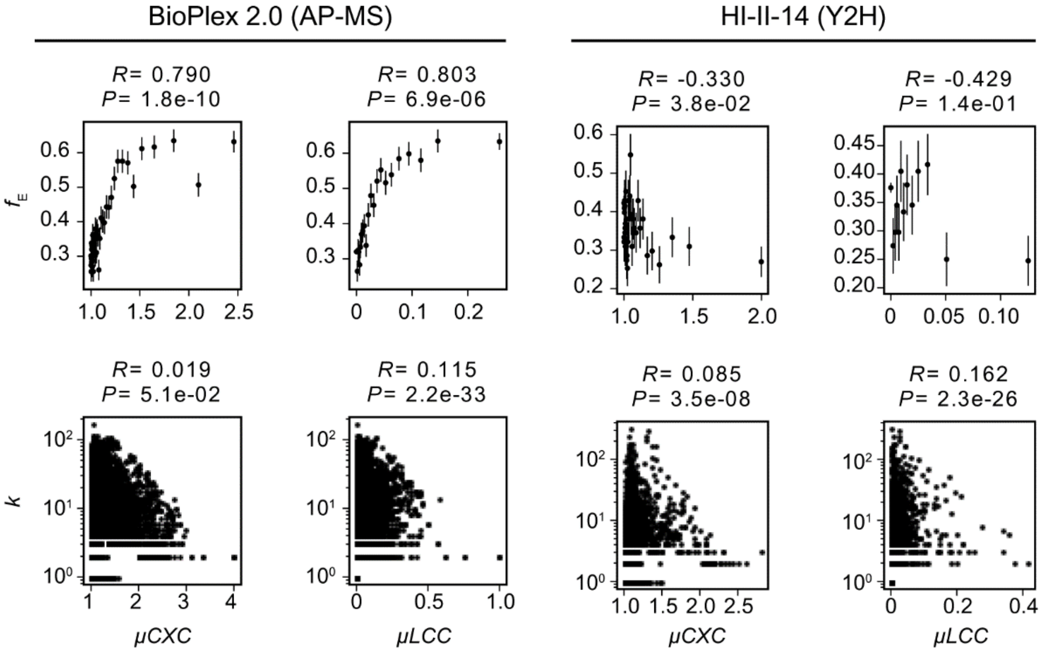

Figure S21. Correlation of the link clustering measures (*μCXC* and *μLCC*) with *k* and f_E in two recent human interactomes.
